# The immunosuppressant drug azathioprine restrains adipogenesis of muscle Fibro/Adipogenic Progenitors from dystrophic mice by affecting AKT signaling

**DOI:** 10.1038/s41598-019-39538-y

**Published:** 2019-03-13

**Authors:** Alessio Reggio, Filomena Spada, Marco Rosina, Giorgia Massacci, Alessandro Zuccotti, Claudia Fuoco, Cesare Gargioli, Luisa Castagnoli, Gianni Cesareni

**Affiliations:** 1 Department of Biology, University of Rome “Tor Vergata”, 00133 Rome, Italy; 2 Fondazione Santa Lucia Istituto di Ricovero e Cura a Carattere Scientifico (IRCCS), 00143 Rome, Italy

## Abstract

Fibro/Adipogenic Progenitors (FAPs) define a stem cell population playing a pro-regenerative role after muscle damage. When removed from their natural niche, FAPs readily differentiate into adipocytes or fibroblasts. This digressive differentiation potential, which is kept under tight control in the healthy muscle niche, contributes to fat and scar infiltrations in degenerative myopathies, such as in Duchenne Muscular Dystrophy (DMD). Controlling FAP differentiation by means of small molecules may contribute to delay the adverse consequences of the progressive pathological degeneration while offering, at the same time, a wider temporal window for gene therapy and cell-based strategies. In a high content phenotypic screening, we identified the immunosuppressant, azathioprine (AZA) as a negative modulator of FAP adipogenesis. We show here that AZA negatively affects the adipogenic propensity of FAPs purified from wild type and *mdx* mice by impairing the expression of the master adipogenic regulator, peroxisome proliferator-activated receptor γ (PPARγ). We show that this inhibition correlates with a decline in the activation of the AKT-mTOR axis, the main pathway that transduces the pro-adipogenic stimulus triggered by insulin. In addition, AZA exerts a cytostatic effect that has a negative impact on the mitotic clonal process that is required for the terminal differentiation of the preadipocyte-committed cells.

## Introduction

Muscle regeneration is governed by a complex cellular crosstalk that is activated after damage^1^. Muscle Satellite Cells (MuSCs) are the main stem progenitors with myogenic potential in the adult muscles^2,3^. In addition, Fibro/Adipogenic Progenitors (FAPs) promote muscle damage resolution by supporting and aiding MuSC proliferation and differentiation^4–6^. However, FAPs are multipotent progenitors and readily differentiate into adipocytes and fibroblasts when *in vitro* cultured. In *in vivo* physiological conditions, this differentiation potential is tightly controlled and restrained. On the other hand, in myopathies, these constraints are progressively lost and FAPs contribute to fat deposition^7^ and scar infiltrates^8^ causing the impairment of the muscle function. Thus, targeting FAPs with small molecules aimed at redirecting their differentiation trajectories, at the expense of the fibro/adipogenic destiny, is a promising strategy to control muscle wasting and degeneration. Inhibitors of the histone deacetylases (HDACi), such as trichostatin A (TSA), target FAPs by inhibiting their adipogenic propensity and unveil a latent myogenic potential via epigenetic reprogramming^9–13^. However, adipogenesis can be triggered by different stimuli acting via the activation of different pathways converging onto the activation of PPARγ. HDACis only target some of these pathways^9–13^. Thus, the importance of identifying new molecules active on FAP differentiation through different mechanisms to be employed to counteract fat infiltrates in myopathies.

The heterogeneous muscle mononuclear cell populations can be separated from the fibres and cultivated *in vitro* where differentiation can be monitored in conditions in which the crosstalk between the different mononuclear populations is allowed to proceed^14^. This experimental system partially recapitulates the *in vivo* cellular context and can be used for screening strategies aimed at selecting molecules affecting differentiation. We used such a complex, albeit robust, *in vitro* system to identify new drugs limiting adipogenesis. By using this approach, we selected and validated the immunosuppressant azathioprine (AZA), the pro-drug of 6-mercaptopurine, as a negative modulator of the adipogenic differentiation. By using purified cell populations, we identified FAPs as the cell population targeted by AZA. AZA treatment impairs FAP adipogenesis by downregulating the transcription factor peroxisome proliferator-activated receptor γ (PPARγ) as a consequence of an attenuation of AKT-mTOR signaling and of a mitotic delay.

## Results

### Azathioprine restrains the intrinsic adipogenic potential of muscle mononuclear cells

**Figure 1:**
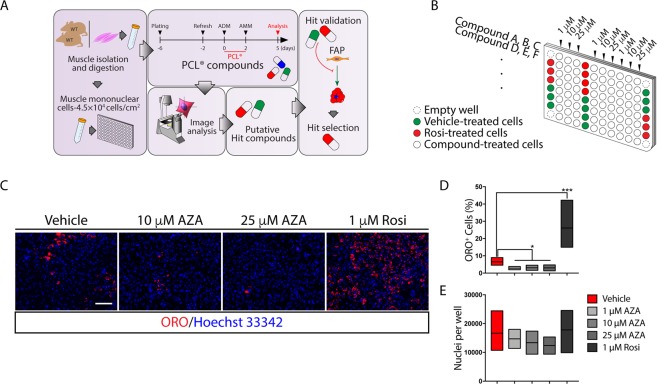
Screening for drugs that limit the muscle mononuclear cell adipogenic potential. (**A**) Flow diagram of the screening strategy. Muscle mononuclear cells were isolated from the hind limbs of wild type mice and seeded at a density of 4.5 × 10^4^ cells/cm^2^ in matrigel coated well. Six-day cultured cells were incubated for 48 hours with adipogenic induction medium (ADM) supplemented with the Prestwick Chemical library® (PCL) compounds. Two days later, drug supplemented media were replaced by drug-free adipogenic maintenance medium (AMM) and cells were cultured for three additional days prior to staining with ORO solution. (**B**) Multiwell screening layout. For the control samples, cells were incubated in 0.5% (v/v) DMSO (green wells). Samples treated with 1 µM rosiglitazone (Rosi) were used as positive controls (red wells). Each compound was tested in a dose-response assay (1, 10, 25 μM) (white wells). Wells at the plate corners just contained medium and were used as negative controls (dashed wells). (**C**) Representative images, at 20x magnification, of Oil Red O (ORO)-staining of the primary screening. Muscle mononuclear cells were incubated in ADM with increasing concentration of azathioprine (AZA) (10, 25 μM) for 48 hours. Cells were then incubated for three additional days in AMM allowing adipocyte differentiation. (**D**,**E**) The floating bar graphs represent the average percentage of ORO-positive cells per field (**D**) and the average number of nuclei per well in each experimental condition (**E**). The statistical significance was estimated by one way ANOVA and defined as *p < 0.05; **p < 0.01; ***p < 0.001 (Vehicle *n* = 9, 1 μM AZA *n* = 2, 10 μM AZA *n* = 2, 25 μM AZA *n* = 2, 1 μM Rosi *n* = 10). Scale bar: (**C**) 100 μm.

Muscle mononuclear cells were isolated from the hind limbs of young C57BL/6J mice (hereafter referred to as wild type) and assessed for their ability to *in vitro* differentiate into different mesodermal lineages by incubating them with BMP-2 (osteogenic), TGF-β (fibrogenic) or with a pro-adipogenic mix containing dexamethasone, 3-isobutyl-1-methylxanthine (IBMX) and rosiglitazone (Rosi). Each differentiation phenotype was assessed by specific staining, demonstrating that the preparation of muscle mononuclear cells had the potential to differentiate into alkaline phosphatase (ALP)-positive osteoblast precursors, α-smooth muscle actin (α-SMA)-myofibroblasts, Oil Red O (ORO)-positive adipocytes or myosin heavy chain (MyHC)-positive myotubes (Supplementary Fig. S1A–I). We used this heterogeneous cell preparation to monitor the perturbations of the adipogenic and/or myogenic program and we developed a medium-scale phenotypic assay (Fig. 1A). A total of 640 molecules, from the Prestwich Chemical Library® (PCL), were tested in a dose-response phenotypic screening by assaying each drug at concentrations of 1, 10, 25 μM (Fig. 1B). Adipogenesis was estimated by monitoring, via automatic image analysis, the percentage of ORO-positive cells. Among all the tested molecules, AZA reduced the percentage of the ORO-positive cells, revealing a significant negative perturbation of the intrinsic adipogenic potential of some cell sub-population(s) within the muscle mononuclear cell preparation (Fig. 1C–E). Here, we report the functional characterization of AZA, an immunosuppressant drug that was identified in the screening as a negative modulator of the adipogenic program of muscle mononuclear cells.

### Azathioprine targets Fibro/Adipogenic Progenitors (FAPs) and limits their *in vitro* intrinsic adipogenic propensity

FAP is the main cell population, within mononuclear cells, which is held responsible for fat and scar infiltrations in myopathies^7^. We asked whether azathioprine could limit the adipogenesis of FAPs derived from wild type and/or *mdx* mice, a model of DMD. We used 1.5-month old mice in our analysis. At this age, *mdx* mice are in their first regenerative window^15^ and isolated FAPs, when *ex vivo* cultured, readily differentiate into adipocytes^12^.

**Figure 2:**
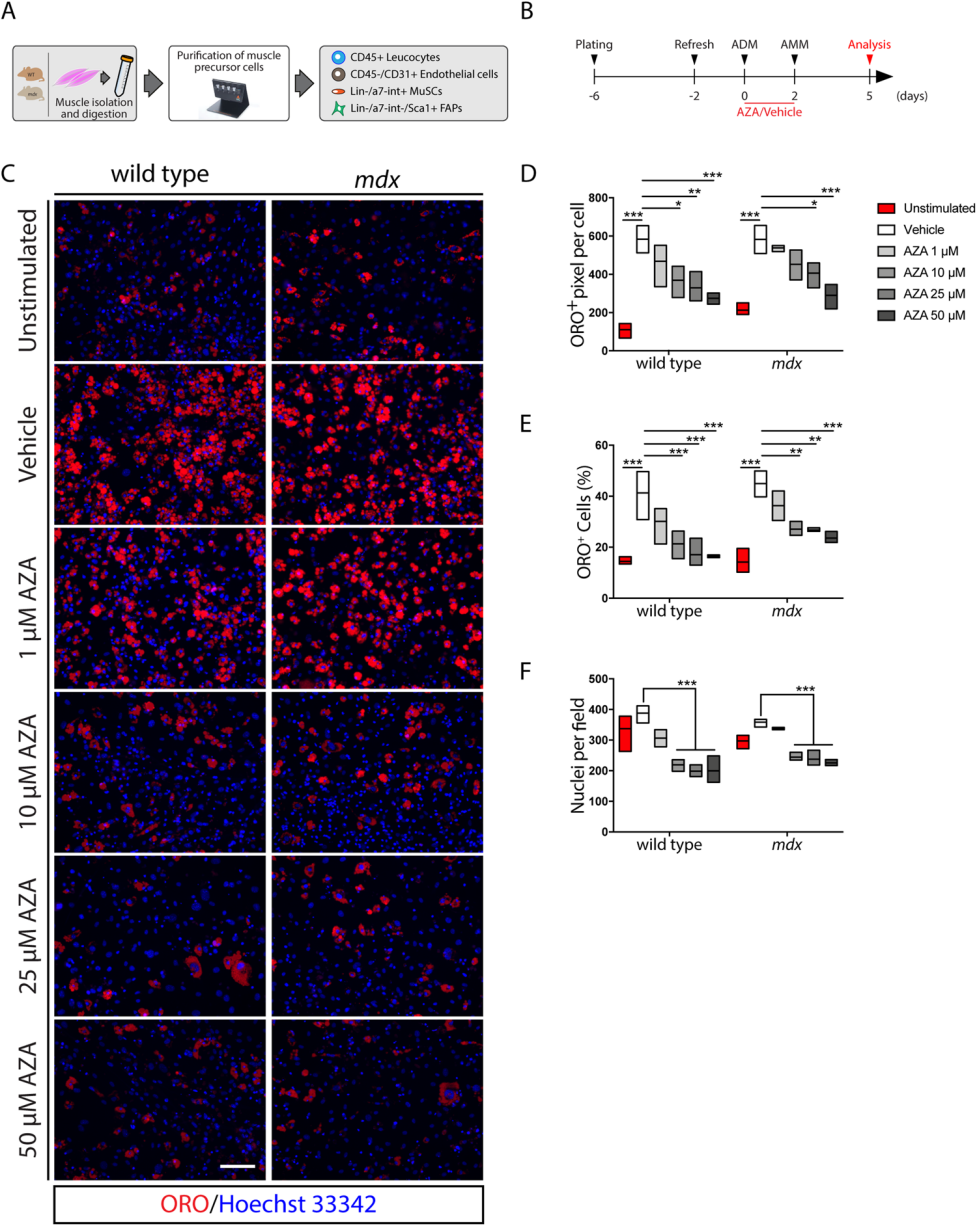
Azathioprine negatively modulates the intrinsic adipogenic potential of FAPs purified from wild type and *mdx* mice. (**A**) Schematic representation of the procedure used to purify muscle precursor cells from the hind limbs of wild type and *mdx* mice. (**B**) Schematic representation of the differentiation protocol applied to induce the adipogenic differentiation of wild type and *mdx* FAPs *in vitro*. (**C**) Representative immunofluorescence (20x magnification) of FAP-derived adipocytes from wild type and *mdx* mice differentiated in the presence of increasing concentrations (1, 10, 25, 50 μM) of AZA or vehicle alone. Adipocytes (red) were stained with ORO solution and nuclei (blue) with Hoechst 33342. (**D**) The floating bar graph represents the average area (expressed in pixel) of ORO staining per cell in each field for each experimental condition. (**E**) Floating bar graph represents the average of the percentage ratio between ORO-positive cells and the number of cells in each field for each experimental condition. (**F**) Floating bar graph represents the average number of nuclei per field in each experimental condition. All experimental data are represented as means of three independent experiments ± SEM. The statistical significance was estimated by one way ANOVA and defined as *p < 0.05; **p < 0.01; ***p < 0.001. Scale bar: (**C**) 100 μm.

FAPs were purified as CD45^−^/CD31^−^/α7-integrin^−^ and Sca-1^+^ cells from wild type and *mdx* mice by magnetic beads-based cell sorting^16^ (Fig. 2A). Sca-1^+^ cells also co-express the Platelet-Derived Growth Factor Receptor-α (PDGFRα) (Supplementary Fig. S2A) which is considered as a distinctive marker of FAPs. Purified FAPs were first incubated for 48 hours with the adipogenic differentiation medium (ADM), at increasing concentrations of AZA (0, 1, 10, 25, 50 μM). Next, cells were cultured for three additional days with the adipogenic maintenance medium (AMM) (Fig. 2B). Differentiation efficiency was assessed by monitoring the ORO-positive pixels in each cell. Incubation in ADM significantly promoted lipid accumulation in wild type as well as *mdx* FAPs when compared to unstimulated cells (Fig. 2C,D). However, ORO staining was significantly reduced in both wild type and *mdx* FAPs when ADM was supplemented with AZA at 10, 25 and 50 μM (Fig. 2C,D). In addition, we also measured the fraction of cells that differentiated into adipocytes. The reduced extent of ORO staining correlates with the decrease in the number of adipocytes upon AZA treatment (Fig. 2C,E). In addition to the adipogenic inhibition, a significant reduction in the cell number was also observed already at 10 μM (Fig. 2F). Overall our data demonstrate that AZA negatively modulates the adipogenic differentiation of wild type and *mdx* FAPs by limiting the acquisition of a terminally differentiated phenotype.

### Azathioprine impairs FAP adipogenesis by attenuating the AKT-mTOR signaling axis and PPARγ expression

**Figure 3:**
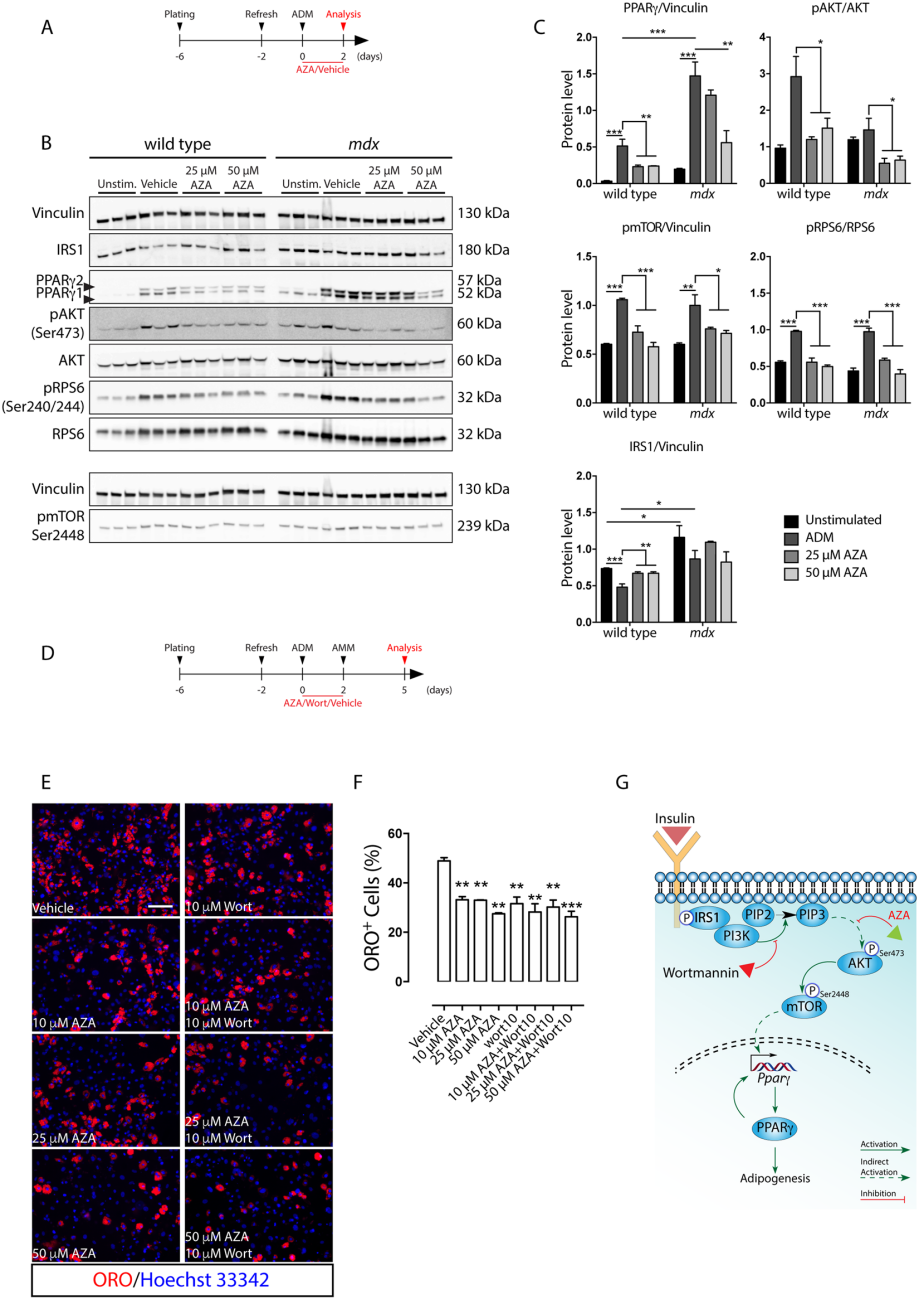
Azathioprine limits PPARγ upregulation by hampering the activation of the AKT-mTOR signaling axis, during wild type and *mdx* FAP adipogenesis. This pathway is similarly impacted by wortmannin. (**A**) Schematic representation of the experimental procedure. (**B**) Immunoblot of wild type and *mdx* FAPs induced to differentiate in the presence of increasing concentration of AZA (0, 25, 50 μM). For each sample 15 μg of protein lysate was electrophoresed on a 4–20% gradient gel and the protein levels of IRS1, PPARγ, pAKT, AKT, pRPS6, RPS6 and pmTOR were revealed with specific antibodies. Vinculin serves as loading and normalization control. (**C**) Bar plots of the densitometric analysis of the gel bands in A. Full-length blots/gels are represented in Supplementary Fig. S6. (**D**) Schematic representation of the experimental procedure applied to induce FAP differentiation in the presence of AZA and/or wortmannin (Wort). (**E**) Representative immunofluorescence of *mdx* FAPs differentiated in the presence of 10 μM wortmannin (Wort) with increasing concentrations (10, 25, 50 μM) of AZA, or vehicle alone. (**F**) Bar plot represents the average of the percentage ratio between ORO-positive cells and the number of cells in each field for each experimental condition. Adipocytes (red) were revealed by ORO staining. Nuclei (blue) were stained with Hoechst 33342. (**G**). Model to describe the inhibition of FAP adipogenesis by AZA and/or wortmannin. All experimental data are represented as means of three independent experiments ± SEM. The statistical significance was estimated by one way ANOVA and defined as *p < 0.05; **p < 0.01; ***p < 0.001.

To investigate the molecular mechanisms perturbed by AZA treatment, we monitored the expression levels of the adipogenesis master gene PPARγ 48 hours after ADM exposure (Fig. 3A). Incubation with ADM induced the expression of both PPARγ isoforms, the increase being more noticeable in *mdx* cells (Fig. 3B,C). Conversely, PPARγ expression was negatively affected when wild type and *mdx* FAPs were induced to differentiate in ADM supplemented with 25 and 50 μM AZA (Fig. 3B,C). Thus, the inhibition of the adipogenesis by AZA parallels a reduction in the expression of PPARγ. We next looked for evidence of perturbations in pathways upstream of PPARγ. We focused on the Protein kinase B (PKB/AKT) pathway, which controls adipogenesis via, at least, two downstream branches, impacting on the Forkhead Transcription Factor 1 (FOXO1) and the Mammalian Target of Rapamycin Complex 1 (mTORC1) that act as negative and positive regulators of PPARγ, respectively^17,18^. AZA and its metabolic derivative 6-mercaptopurine (6-MP) have been shown to reduce the phosphorylation levels of AKT^19,20^. Compared to unstimulated FAPs, the incubation with ADM for 48 hours promoted the activation of AKT-mTORC1 signaling, as revealed by monitoring the phosphorylation of activating residues of AKT (Ser473), mTOR (Ser2448) and Ribosomal Protein S6 (RPS6) (Ser240/244) (Fig. 3B,C). The downregulation of the insulin receptor substrate 1 (IRS1), possibly as a consequence of a negative feedback mediated by Ribosomal Protein S6 Kinase (S6K), is consistent with the sustained activation of the AKT-mTOR axis in wild type as well as in *mdx* FAPs after 48 hours of exposure to ADM (Fig. 3B,C). By contrast, when FAPs were induced to differentiate in the presence of 25 or 50 μM AZA, we observed a decrease in the activation of the AKT-mTOR axis (Fig. 3B,C). Consistent with the hypothesis that AZA inhibits adipogenesis by downregulating the PI3K/AKT/mTOR axis, cotreatment with AZA and wortmannin (Fig. 3D), an inhibitor of Phosphatidylinositide 3-Kinase (PI3K), did not show any additive nor synergic effect (Fig. 3E,F), suggesting that both drugs affect the same signaling pathway (Fig. 3G). Hence, we conclude that, in FAPs undergoing *in vitro* adipogenesis, AZA impairs the upregulation of PPARγ and that this negative modulation correlates with the blunted activation of AKT-mTOR signaling.

### AZA interferes with insulin-mediated adipogenesis of dystrophic FAPs

The observation that the inhibition of the adipogenesis mediated by AZA is accompanied by a decrease in AKT activation prompted us to formulate the hypothesis that AZA interferes with adipogenesis induced by insulin, the pro-adipogenic hormone included in the adipogenic cocktail. To test this hypothesis, we reduced the complexity of the pro-adipogenic mix by removing dexamethasone and IBMX and we asked whether AZA would still be able to negatively modulate the adipogenesis of *mdx* FAPs in these conditions.

**Figure 4:**
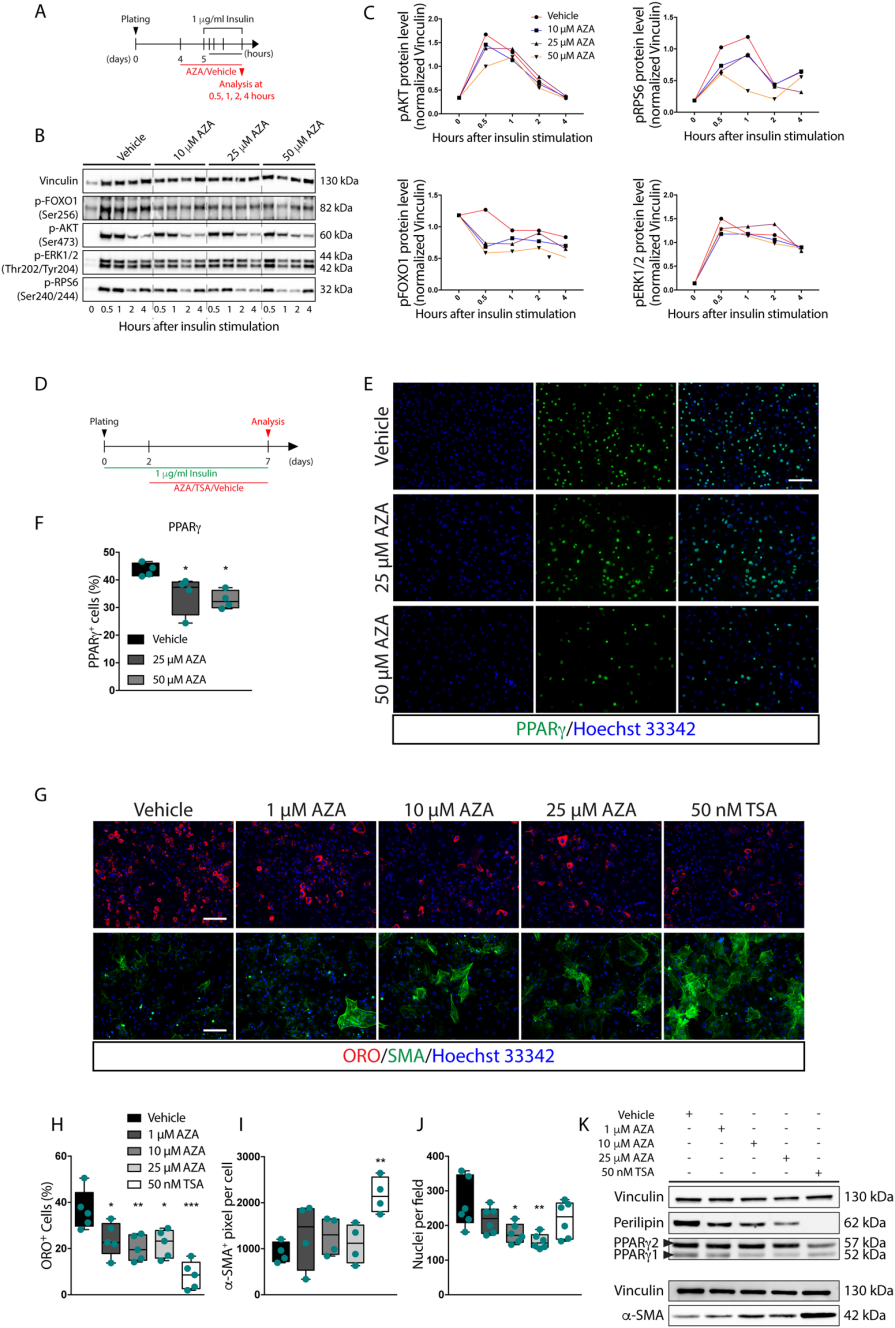
Azathioprine desensitizes *mdx* FAPs to insulin. (**A**) Schematic representation of the differentiation protocol used to stimulate insulin signaling of *mdx* FAPs in the presence of 1 μg/ml of insulin. (**B**) For each sample 10 μg of the whole protein lysate was electrophoresed on a 4–20% gradient gel and the protein levels of pAKT, pRPS6, pFOXO1 and pERK1/2 were revealed with specific antibodies. Vinculin serves as loading and normalizing control. Full-length blots are represented in Supplementary Fig. S7. (**C**) Densitometric analysis of the protein levels of pAKT, pRPS6, pFOXO1, pERK1/2, normalized over vinculin. (**D**) Schematic representation of the protocol adopted to induce adipogenic differentiation of *mdx* FAPs in the presence of 1 μg/ml of insulin. (**E**) Representative immunofluorescence, 20x magnification, of differentiated *mdx* FAPs immunostained using an anti-PPARγ antibody (green) after treatment either with 25 and 50 μM of AZA or vehicle alone. Nuclei (blue) were stained with Hoechst 33342. (**F**) Box plot represents the average of the percentage ratio between PPARγ-positive cells and the number of cells in each field for each experimental condition. (**G**) Representative immunofluorescence of *mdx* FAPs differentiated either in the presence of increasing concentrations (1, 10, 25, 50 μM) of AZA, 50 nM TSA or vehicle alone. Adipocytes (red) were revealed by ORO staining, while myofibroblasts (green) were revealed using an anti-α-SMA antibody. Nuclei (blue) were stained with Hoechst 33342. (**H**) Box plot represents the average of the percentage ratio between ORO-positive cells and the number of cells in each field for each experimental condition. (**I**) Box plot represents the average extent (expressed in pixel) of α-SMA-positive area per cell in each field for each experimental condition. (**J**) Box plot represents the average number of nuclei per field for each experimental condition. (**K**) Representative immunoblot in which, for each sample, 30 μg of the protein lysate was electrophoresed on a 10% SDS-PAGE and the protein levels of perilipin, PPARγ isoforms and α-SMA were revealed with specific antibodies. Vinculin serves as loading and normalizing control. Full-length blots/gels are represented in Supplementary Fig. S8. All experimental data are represented as means of at least three independent experiments ± SEM. The statistical significance was estimated by one way ANOVA and defined as *p < 0.05; **p < 0.01; ***p < 0.001. Scale bar: (E, G) 100 μm.

FAPs were isolated from *mdx* mice and stimulated with 1 μg/ml of insulin in the presence of AZA (Fig. 4A). As observed for the medium with the complete pro-adipogenic cocktail, AZA reduced the insulin mediated activation of the AKT-mTOR pathway. This inhibition was already evident at an early time point (0.5 hours) (Fig. 4B,C). By contrast, the phosphorylation of Extracellular signal-regulated kinase 1/2 (ERK1/2), was similar in both vehicle and AZA-treated samples (Fig. 4B,C). These data confirm that AZA interferes with the upstream events that lead to AKT phosphorylation and activation in response to insulin. As a consequence, mTOR activity is dampened as revealed by the reduced phosphorylation of RPS6 and FOXO1.

The medium supplemented with 1 μg/ml of insulin was sufficient to induce FAP adipogenesis after seven days^16^ (Fig. 4D,G). However, when cells were exposed to 25 and 50 μM AZA, the treatment significantly reduced the number of PPARγ-positive FAPs at all tested concentrations (Fig. 4E,F). In comparison, 50 nM Trichostatin A (TSA), an inhibitor of FAP adipogenesis^12,13^, almost completely blocked FAP differentiation (Fig. 4G,H,K and Supplementary Fig. S2B–D). The reduced adipogenesis upon AZA exposure (Fig. 4G,H) parallels the reduced expression of PPARγ1 (Fig. 4K and Supplementary Fig. S2B,F,G) and Perilipin (Fig. 4K and Supplementary Fig. S2D). Conversely, PPARγ2 isoform was hardly affected by AZA (Fig. 4K and Supplementary Fig. S2C,F,H). Also in insulin differentiating FAPs, AZA treatment significantly affects cell number (Fig. 4J). Noteworthy, 25 μM AZA as well as 50 nM TSA down regulated the expression of PPARγ and adiponectin (Adipoq) at transcriptional level without altering the expression of the transcription factor CCAAT/Enhancer Binding Protein β (C/EBPβ) (Supplementary Fig. S2I). In addition, immunofluorescence analysis demonstrated that AZA did not affect the spontaneous differentiation of FAPs into α-SMA-positive myofibroblasts, while treatment with 50 nM TSA significantly enhances α-SMA expression in FAPs isolated from *mdx* mice (Fig. 4G,I) as confirmed by western blot analysis (Fig. 4K and Supplementary Fig. S2E). Consistently, by looking at the transcription of some fibrogenic genes (*s100a4*, *Col1a2*, *Col6a1* and *Fn1*), we did not observe any significant perturbation upon treatment with 25 μM AZA or 50 nM TSA (Supplementary Fig. S2I). Moreover, 48 hours of drug treatment, with increasing concentrations of AZA and 50 nM TSA, did not affect myogenin and MyHC expression (Supplementary Fig. S3A–I). Hence, AZA, at concentrations that inhibit FAP adipogenesis neither did significantly affect the growth rate of *mdx* MuSCs, nor the expression kinetic of myogenin and MyHC (Supplementary Fig. S3J–L), thus revealing a differential sensitivity of FAP and MuSC differentiation^21^ to AZA. The concentrations of AZA and TSA, which were active in blocking FAP adipogenesis, were also able to inhibit the accumulation of intracellular lipids in differentiating 3T3-L1 preadipocyte cell line, this inhibition being most evident when the treatment was carried out for 48 hours in ADM (Supplementary Fig. S4A–D). Consistently, the inhibitory effect of AZA on 3T3-L1 differentiation correlates with the down-regulation in the expression of PPARγ and Perilipin (Supplementary Fig. S4E–G). By monitoring the activating phosphorylation sites of AKT and RPS6, it was evident that the blunted activation of the AKT-mTOR axis exclusively occurred in the presence of the anti-adipogenic concentrations of AZA, while remaining unaffected upon treatment with 50 nM TSA (Supplementary Fig. S4H–J).

### Azathioprine affects the mitotic rate of differentiating FAPs

**Figure 5:**
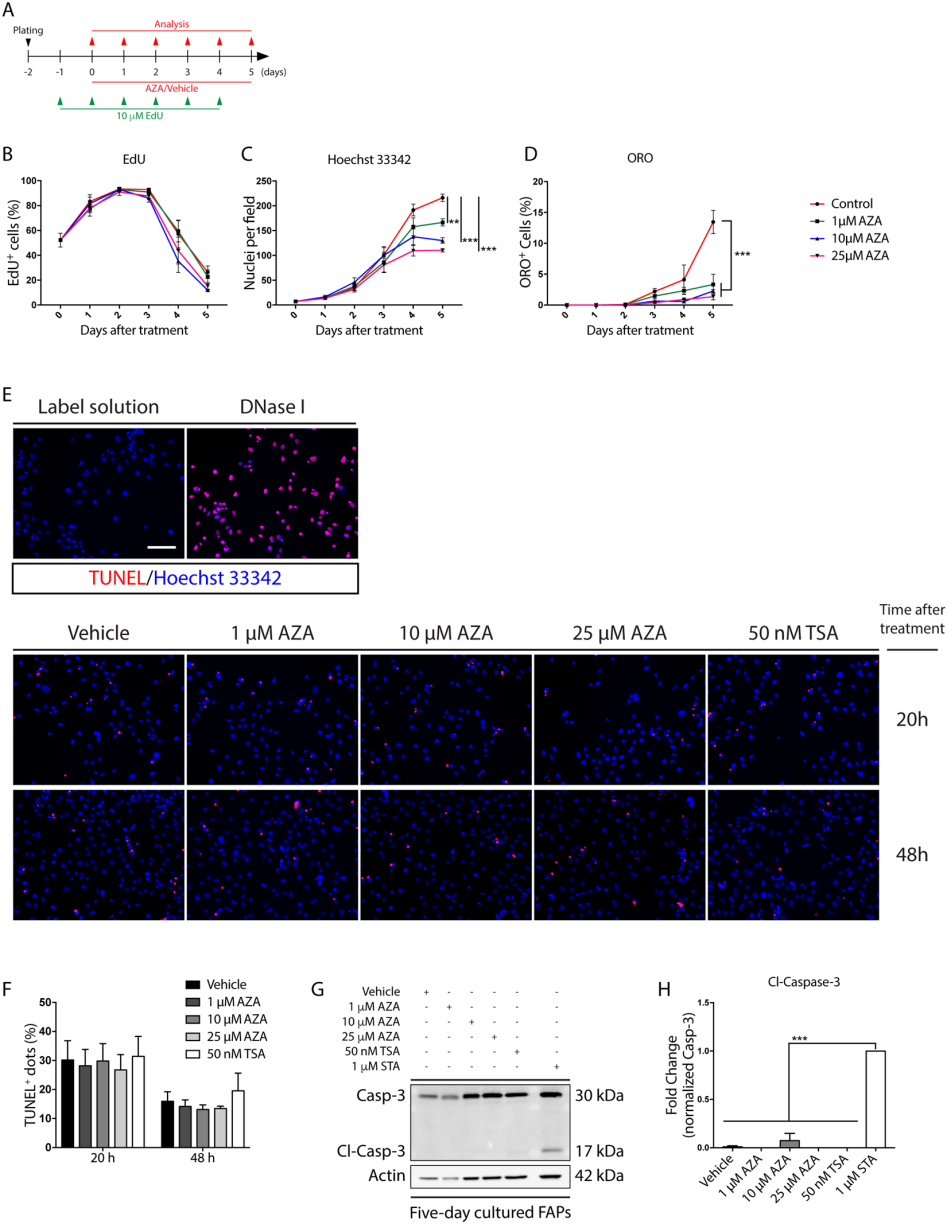
Azathioprine delays FAPs growth without inducing apoptosis. (**A**) Experimental plan for EdU labelling of *in vitro* cultured *mdx* FAPs. (**B**) The average ratio of EdU-positive FAPs over the total cell number in each condition. (**C**) Time course of the average number of cells per field. (**D**) The average ratio of ORO-positive cells over the total cells in each condition. (**E**) Representative TUNEL assay images of *mdx* FAPs immunostained 20 and 48 hours after AZA (0, 1, 10, 25 μM) and TSA (50 nM) treatment. A sample stained with the label solution alone and a sample pre-treated with DNase I were used as negative and positive control, respectively. Nuclei (blue) were stained with Hoechst 33342. (**F**) The bar plot represents the average of the ratio between TUNEL-positive dots and the number of cells in each field for each experimental condition. The statistical significance, was estimated by two way ANOVA and defined as *p < 0.05; **p < 0.01; ***p < 0.001. (**G**) Representative immunoblot in which for each sample 15 μg of protein lysate was electrophoresed on a 15%^®^ SDS-PAGE and the protein levels of the cleaved and the total form of Caspase-3 were simultaneously revealed with a specific antibody. Actin serves as loading control. Full-length blots are represented in Supplementary Fig. S9. (**H**) The bar plot represents the densitometric analysis of the ratio between the cleaved Caspase-3 over the total signal of caspase-3. 1 μM staurosporine (STA) was used as positive control. All experimental values are represented as means of at least three independent experiments ± SEM and the statistical significance, was estimated by one way ANOVA and defined as *p < 0.05; **p < 0.01; ***p < 0.001. Scale bar: (**E**) 100 μm.

We next investigated the reasons behind the cell number reduction upon AZA treatment. We first evaluated the mitotic rate at different time points after AZA treatment, by incubating *mdx* FAPs with 5-ethynyl-2′-deoxyuridine (EdU) for 24 hours (Fig. 5A). At time 0, only 50% of the cells entered the cell cycle, while at day 3 more than 80% were actively replicating without any significant differences between treated and control cells. At days 4 and 5, however, the mitotic rate decreases and this decrease is more noticeable in azathioprine-treated cells (Fig. 5B). The decrease in mitotic rate is paralleled by a significant reduction of the proliferation rate that plateaus at day 4 and day 5 when the untreated cells differentiate (Fig. 5C,D). The reduced cell number upon AZA treatment was not caused by apoptosis (Fig. 5E–H).

Cell cycle progression is crucial for allowing committed cells to acquire a terminally adipogenic phenotype^22^. Thus, we checked whether AZA could affect the mitotic clonal expansion (MCE). To address this point, we monitored the cell cycle progression of 3T3-L1 preadipocytes that synchronously enter the cell cycle upon adipogenic induction. Cell cycle analysis up to 48 hours following induction in adipogenic medium revealed a dose-dependent cell cycle delay in the G1/S phase transition when cells were exposed to AZA (Supplementary Fig. S5A,B). TSA, on the other hand, completely blocked cell cycle entry of 3T3-L1 preadipocytes (Supplementary Fig. S5A,B). Overall these results are consistent with the fact that AZA and TSA negatively affect the adipogenic program by dampening or preventing the MCE progression.

## Discussion

In Duchenne Muscular Dystrophy (DMD), mutations in the dystrophin gene predispose for a progressive degeneration of the striated muscles as a consequence of the depletion of the regenerative potential of Muscle Satellite Cells (MuSCs)^23,24^. At the same time, muscle function is compromised by progressive myofibre wasting together with fibrosis and fatty infiltrations. Although the multipotency of MuSCs remains ill-defined and controversial^25,26^, the massive ectopic tissue depositions observed in myopathies are unlikely to be a consequence of cell autonomous or non-autonomous mechanisms that prompt MuSCs toward non-myogenic destinies. Conversely, FAPs have a strong propensity to differentiate into adipocytes and fibroblasts^4,5^. In a physiological context, signals provided by the niche activate pathways (SHH, TNFα, NOTCH) that contrast FAP adipogenic potential^4,5,8,27,28^ while, at the same time, instructing FAPs to contribute to muscle homeostasis and regeneration^4–6^. In myopathies, the attenuation^8^ of these physiological constraints and/or an acquired FAP insensitivity^28^ to the same constraints, release FAP fibro/adipogenic potential. Eventually fat infiltrations and fibrosis alter the functional and structural architecture of the muscle^7^. For these reasons, FAPs are a promising cellular target for pharmacological therapies finalized at blocking disease progression^8,12,21^. Hence the importance of providing a wide range of molecules altering FAP differentiation trajectories. To address this issue, we developed a cell based phenotypic assay designed to identify “perturbagens” that negatively modulate the adipogenic program in muscle progenitor cells. Following the primary screening, we performed a stringent validation of each anti-adipogenic candidate drug on purified FAPs. By using this approach, we identified azathioprine as a drug that negatively perturbs the intrinsic adipogenic trajectory of FAPs.

Azathioprine (AZA), one of the oldest immunosuppressive drugs currently in use, is a member of the thiopurine family. Tested in a randomized trial in DMD patients as an alternative immunosuppressant, AZA did not exert any physical benefit when compared to prednisone^29^, although muscle biopsies showed comparable immunohistological ameliorations^30^. While the primary mechanism underlying AZA pharmacological activity can be imputed to its interference with DNA synthesis^31,32^, alternative biological processes have been implicated in the drug mechanism of action^19,20,33–37^.

In the present study, we demonstrate that AZA limits the adipogenic propensity of FAPs at concentrations that are compatible with those reached in the blood of patients treated with therapeutic doses^19,38–40^. The inhibition of adipogenesis correlates with a decrease in the expression of the master adipogenic gene, PPARγ. By dissecting the signaling pathway upon AZA treatment, we demonstrated that the impaired expression of PPARγ correlates with the blunted activation of the PI3K/AKT/mTOR axis. In this context, by reducing the complexity of the adipogenic cocktail, we could clarify that AZA selectively targets the insulin pathway by down-regulating the AKT-mTOR signaling axis, mainly impairing PPARγ1 expression and resulting in the failure to acquire an adipogenic phenotype. Similarly, the inhibition of PI3K, using wortmannin, was able to negatively affect FAP adipogenesis and the cotreatment with AZA did not strengthen this inhibition, suggesting that both molecules act on the same node, the AKT-mTOR pathway. Thus, AZA acts through a mechanism that is different from that of the HDAC inhibitor TSA. TSA blocks the adipogenic program of FAPs through a mechanism that is independent from AKT-mTOR signaling^13^, as observed in 3T3-L1 pre-adipocytes. On the other hand, AZA negatively modulates the adipogenic differentiation of FAPs without affecting the expression of key fibrogenic genes. In addition, the myogenic differentiation of primary MuSCs is not affected by AZA treatment. Finally, we questioned whether cytostatic/cytotoxic effects could contribute to the observed anti-adipogenic activity. The concentrations of AZA used in our experiments did not cause apoptosis, while EdU-labelling of *in vitro* cultured FAPs highlighted a reduction in the fraction of cells incorporating EdU starting at day 4 after drug exposure. To further examine the cell cycle perturbation caused by AZA, we used synchronously growth arrested 3T3-L1. This type of analysis clearly demonstrated that the antiadipogenic concentrations of AZA impair the fine-tuned cell cycle progression along the mitotic clonal expansion phase (MCE)^22^. Surprisingly, 50 nM TSA completely prevented the MCE in 3T3-L1. This may reflect an additional layer of regulation through which TSA affects the adipogenic program of mesenchymal progenitors, in addition to the already described epigenetic modulation^13^.

In summary, we establish the muscle mononuclear cell preparation as a heterogenous system suitable for phenotypic screening aimed at recapitulating the *in vivo* cell complexity of the muscle tissue. We also provide for the first-time evidence for the effectiveness of azathioprine in limiting the *in vitro* adipogenic differentiation of a pure (over 90%) FAP preparation. However our FAP cultures contained less than 7% Sca1^−^ and/or PDGFRα^−^ cells, these cells are not myogenic as no myotube were observed in condition that favour FAP differentiation. Although this no-FAP cells have not been characterized in this study, it is unlikely that these cells could play a confusing role in our experiments. Our findings relate to the effect of the drug azathioprine on the differentiation of isolated primary cells when FAPs readily differentiate into adipocytes in the absence of the plethora of signals (SHH, TNFα, NOTCH) that *in vivo* modulate their differentiation potential^4,5,8,27,28^. Proving that azathioprine has any effect in limiting adipogenesis *in vivo* in the presence of such a redundancy of control mechanisms would offer an alternative strategy for limiting fat infiltrates in the pathogenesis of muscular dystrophies (MDs). This conclusion however would require careful pharmacological studies. By comparing the molecular underpinnings of the mechanisms underlying the anti-adipogenic effects of AZA and TSA, we conclude that they act by different mechanisms and as such could show a synergic effect when co-administered *in vivo*.

## Materials and Methods

### Muscle mononuclear cell purification

C57BL/6J and C57BL10ScSn-Dmd^mdx^/J mice (hereafter referred to as *wild type* and *mdx* respectively) were purchased from the Jackson Laboratories. Rodents were bred and maintained according to the standard procedure in the animal facility. All experimental procedures were conducted according to the rules of good animal experimentation I.A.C.U.C. n°432 of March 12-2006 and under ethical approval released on 11-12-2012 from the Italian Ministry of Health, protocol #20/01-D.

The muscle mononuclear cells employed for the screening were isolated from 25-day old wild type mice, while experiments on FAPs and MuSCs were performed using 45-day old wild type and *mdx* mice. Briefly, hind limbs were surgically removed and then minced in Hank’s Balance Salt Solution (HBSS Gibco, catalog 14025-092) supplemented with 100 U/ml penicillin/streptomycin (P/S) (Life Technologies, catalog 15140122) and 0.2% bovine serum albumin (BSA) (AppliChem, catalog A1391). The homogeneous muscle tissue preparation was digested with an enzymatic mixture containing 2 µg/µl collagenase A (Sigma-Aldrich, catalog 11088793001), 2.4 U/ml dispase II (Roche, catalog 04942078001) and 10 µg/ml DNase I (Sigma-Aldrich, catalog 11284932001) in Dulbecco’s Phosphate Buffered Saline (DPBS, Biowest, catalog L0625-500) with calcium and magnesium (Gibco, catalog 14040). The digestion period was for one hour at 37 °C in gentle shaking. The tissue suspension underwent three consecutive filtrations through 100, 70 and 40 µm cell strainers (DB Falcon, catalog 352360, 352350, 352340, respectively). Red blood cells were lysed in RBC Lysis Buffer (Santa Cruz Biotechnology, catalog sc-296258) avoiding any contamination by the erythroid lineage. Finally, cells were centrifuged at 800 × *g* and resuspended in Growth Medium (GM) composed by 20% fetal bovine serum (Euroclone, catalog ECS0180L), 10 mM Hepes (Sigma-Aldrich, catalog H0887), 1 mM sodium pyruvate (Sigma-Aldrich, catalog 68636), 100 U/ml P/S (Life Technologies, catalog 15140122) in high glucose Dulbecco’s Modified Eagle’s Medium (DMEM) GlutaMAX™ (Life Technologies, catalog 61965-059). Isolated cells were seeded at a density of 4.5 × 10^4^ cells/cm^2^ on matrigel (BD, catalog 356234) coated 96-well. Alternatively, muscle mononuclear cells were magnetically sorted.

### Purification of Muscle Satellite Cells (MuSCs) and Fibro/Adipogenic Precursors (FAPs)

Muscle Satellite Cells (MuSCs) and Fibro/Adipogenic Progenitors (FAPs) were purified from wild type and *mdx* mice using the magnetic beads cell sorting MACS purification system (Miltenyi). Briefly, freshly isolated muscle mononuclear cells were resuspended in Magnetic Beads Buffer (MBB, 0.2% BSA, 2 mM EDTA in PBS 1X) and filtered through a 30 µm Pre-Separation Filter (Miltenyi Biotec, catalog 130-041-407) in order to remove large particles from the single cell suspension. The single cell suspension was incubated in MBB with anti-CD31 microbeads mouse (Miltenyi Biotec, catalog 130-097-418) and anti-CD45 microbeads mouse antibodies (Miltenyi Biotec, catalog 130-052-301). Magnetically labeled cells were positively selected through the MS column (Miltenyi Biotec, catalog 130-042-201) depleting the CD31/CD45-positive cells from the cell suspension. The negative lineage (Lin-) cells, collected from the flow-through, were resuspended in MBB and incubated with mouse anti α7-integrin microbeads (Miltenyi Biotec, catalog 130-104-261) and positively selected as α7-integrin-positive Muscle Satellite Cells (MuSCs). Lastly, unlabelled cells were incubated in MBB with anti-Sca1 mouse microbeads (Miltenyi Biotec, catalog 130-106-641) and positively selected as Sca1-positive Fibro/Adipogenic Progenitor (FAPs) cells. The sorting procedures and the labelling procedures using the micro-beads conjugated antibodies were performed according to the manufacturer’s instructions.

### MuSCs and FAPs, culture conditions and treatments

Freshly sorted wild type and *mdx* MuSCs were pre-plated in pre-warmed Satellite Cell-Growth Medium (SC-GM) composed by 20% FBS, 10% donor horse serum (Euroclone, ECS0090D), 2% chicken embryo extract (Seralab, CE-650-J), 10 mM Hepes, 1 mM sodium pyruvate, 100 U/ml P/S in high glucose DMEM GlutaMAX™ for 2 hours to reduce fibroblasts contamination. MuSCs were seeded at the cell density of 1.5 × 10^4^ cell/cm^2^ in matrigel-coated wells or dishes. Freshly purified MuSCs were cultured for two days and then incubated for the subsequent 48 hours with vehicle (DMSO), azathioprine (AZA) and trichostatin A (TSA) at the experimental concentrations. After this period, drug-supplemented media were removed and MuSCs cultured for three additional days in Satellite Cell-Differentiation Medium (SC-DM) composed by 2% donor horse serum, 0.5% chicken embryo extract, 10 mM Hepes, 1 mM sodium pyruvate, 100 U/ml P/S in high glucose DMEM GlutaMAX™.

Freshly purified wild type and *mdx* FAPs were resuspended in pre-warmed GM and seeded at the cell density of 1.5 × 10^4^ cell/cm^2^. Four days after plating, the GM was fully refreshed and cells cultured for two additional days before differentiation. The adipogenic differentiation was induced incubating FAPs with the adipocyte differentiation medium (ADM) for two days followed by three additional days in adipocyte maintenance medium (AMM). For *in vitro* treatments, AZA, TSA and rosiglitazone were added at the appropriate concentrations at the onset of differentiation concurrently with the ADM exposure.

Alternatively, freshly purified *mdx* FAPs were directly resuspended and cultured in pre-warmed GM supplemented with 1 µg/ml insulin. After two days, the medium was removed and FAPs incubated in the presence of appropriate concentrations of AZA and TSA and cultured for five additional days in the insulin enriched GM.

### Library compounds

The commercial Prestwick Chemical Library®^®^ (http://www.prestwickchemical.com), containing 1280 FDA-approved drugs, was adopted for the drug screening. All compounds were pre-dissolved in 100% dimethyl sulfoxide (DMSO) at the final concentration of 10 mM and stored at −20 °C. 640 molecules were tested in a dose response high-content screening and each molecule was tested at 1, 10 and 25 µM.

### Chemicals

Azathioprine was purchased from Selleckchem (http://www.selleckchem.com) in a powder form or in DMSO-dissolved form (catalog S1721). Trichostatin A (catalog S1045) and wortmannin (catalog S2758) were purchased from Selleckchem in a powder form.

Chemicals were reconstituted in 100% DMSO Hybri-Max™ (Sigma-Aldrich, catalog D2650) according to the manufacturer’s recommendations.

### Immunoblotting

FAPs, MuSCs and 3T3-L1 cells, were washed in PBS 1X and lysed in ice cold lysis buffer (150 mM NaCl, 50 mM Tris-HCl pH 7.5, 1% Nonidet P-40, 1 mM EDTA, 1% Triton X-100) supplemented with 1 mM ortovanadate, 1 mM NaF, protease inhibitor mixture 1X (Sigma-Aldrich, Catalog P8340), inhibitor phosphatase mixture II 1X (Sigma-Aldrich, catalog 5726) and inhibitor phosphatase mixture III 1X (Sigma-Aldrich, catalog P0044) prior to use. Cell lysates were incubated in ice for 30 minutes and then separated at 15,500 × g in a refrigerated centrifuge. Protein concentration was estimated using Bradford reagent (BioRad, catalog 500-0006). Total protein extracts were resolved by 10%, 15% SDS-PAGE or 4–20% Bio-Rad CRITERION® gradient gel according to the needs. Proteins were transferred to Trans-Blot^®^ Turbo^™^ mini or midi nitrocellulose membranes (Bio-Rad, catalog 1704156–1704157) using a Trans-Blot^®^ Turbo™ transfer System (Bio-Rad) and the non-specific binding were saturated for 1 hour at room temperature (RT) in blocking solution (5% milk, 0.1% Tween-20 in 1X Tris Buffered Saline). Saturated membranes were incubated with specific primary antibodies diluted in blocking solution according to the manufacturer’s instruction. The binding of primary antibodies was revealed using host-specific secondary antibodies. Chemiluminescent detection was performed using Clarity™ Western ECL Blotting Substrates (Bio-Rad, catalog 1705061) and the Fujifilm Las-3000 imaging system. Bands densities were quantified using ImageJ. Brightness and contrast of each blot was adjusted using the “Auto Contrast” function in Adobe Photoshop. The complete list of the antibodies used in this study is reported in Supplementary Table S1.

### Immunofluorescence

Cells were fixed in 2% PFA for 10 minutes at RT and permeabilized in 0.5% Triton X-100. Nonspecific binding sites were saturated for 1 hour at RT in blocking solution (10% FBS, 0.1% Triton X-100 in PBS 11X). Primary antibodies were diluted in blocking solution and incubated for 1 hour at RT. Labeled cells were washed four times with 0.1% Triton X-100 in PBS 1X and incubated in the dark for 30 minutes at RT with the host-specific secondary antibodies diluted in blocking solution. Cells were washed four times with 0.1% Triton X-100 in PBS 1X and counterstained for 5 minutes at RT with 1 mg/ml Hoechst 33342 (Thermo Fischer Scientific, catalog 3570) in 0.1% Triton X-100 in PBS1X. Primary and secondary antibodies were diluted according to the manufacturer’s recommendations. The complete list of the antibodies used in this study is reported in Supplementary Table S1.

### Oil Red O staining

Oil Red O (Sigma Aldrich, catalog O0625) stock solution was prepared according to the manufacturer’s recommendations. Fixed cells were washed twice with 1X PBS and incubated for 20 minutes with Oil Red O in a 3:2 ratio with ultra-pure water. Stained cells were washed twice with 1X PBS and counterstained using Hoechst 33342. Oil Red O stained cells were acquired via fluorescence microscopy.

### TUNEL assay

The detection of the cell death at single cell level was performed by immunofluorescent analysis using the *In-Situ* Cell Death Detection Kit, TMR Red (Roche, catalog 12156792910) following the manufacturer’s recommendations. Micrographs were acquired at 20x magnification.

### EdU incorporation assay

For the proliferation assay cells were seeded at the concentration of 6.0 × 10^3^ cells/cm^2^ and incubated the day prior fixation in the presence of 10 μM of 5-ethylnyl-2′-deoxyuridine (EdU) (Thermo Fisher, catalog C10337). Click-iT® reaction was performed according to the manufacturer’s instructions. Micrographs were acquired at 20x magnification.

### Images acquisition and analysis

The immunofluorescences resulting from the medium-scale screening and low throughput experiments were automatically acquired via LEICA fluorescent microscope (DMI6000B). Twenty-five images per well were acquired at 20x or 10x magnification. All screening images were automatically analyzed in unbiased fashion using CellProfiler^41^ through a dedicated pipeline. Briefly, the algorithm identified Hoechst 33342-stained nuclei as primary objects and then ORO-positive or myogenin-positive objects as secondary objects in their respective channels. The compound effects were scored using the standard score (Z-score) as discriminant. A non-stringent selection of the hit compounds was conducted using a one-standard deviation cutoff from the mean. All images were supervised by manual checking.

### Cell differentiation measurements

Adipogenic differentiation of FAPs was estimated in unbiased fashion using CellProfiler by estimating the average ORO-stained area (expressed in pixels) for each field and normalized over the average number of cells per field. Alternatively, adipogenic differentiation was evaluated by calculating the average number of adipocytes and normalized over the average number of cells per field. Positive objects were manually scored using ImageJ by two independent collaborators. Similarly, the fraction of PPARγ expressing FAPs was estimated in unbiased fashion using CellProfiler. Adipogenic differentiation of 3T3-L1-derived adipocytes was calculated by eluting into pure isopropanol the ORO solution from the ORO-stained samples and the OD_495_ was spectrophotometrically measured.

Fibrogenic differentiation and osteogenic differentiation were estimated using CellProfiler by estimating the SMA or ALP-stained area for each field (expressed in pixels). Values were normalized over the average number of cells per field.

Myogenic differentiation of MuSCs was evaluated as ratio between the average number of myogenin-positive cells for each field over the average number of cells per field. Alternatively, myogenic potential of MuSCs was scored by calculating the fusion index determined as the ratio percentage between the average number of nuclei included into MyHC-expressing cells (containing at least three nuclei) over the average number of nuclei per field. Myotubes diameter was estimated by measuring the myotube thickness at ¼, ½, and ¾ and averaged. At least 10 myotubes for each condition were considered.

The percentage of TUNEL and EdU-positive cells was estimated in unbiased fashion through a dedicated pipeline using CellProfiler. The average number of TUNEL and EdU-positive cells for each field was normalized over the average number of cells per field.

### Statistical analysis

Data are reported as mean ± SEM of at least three independent experiments unless otherwise mentioned. Statistical significance between two groups was estimated using the unpaired Student’s *t*-test assuming two-tailed distribution with a significance defined as *p < 0.05; **p < 0.01; ***p < 0.001. Multiple comparisons between three or more groups were performed using one-way or two-way ANOVA with Dennett’s or Tukey’s test and the statistical significance defined as *p < 0.05; **p < 0.01; ***p < 0.001. All statistical analysis was performed using Prism 7 (GraphPad).

## Supplementary information

The immunosuppressant drug azathioprine restrains adipogenesis of muscle Fibro/Adipogenic Progenitors from dystrophic mice by affecting AKT signaling

## Data Availability

The datasets generated during and/or analysed during the current study are available from the corresponding author on reasonable request.
