# Supplementary material for: The immunosuppressant drug azathioprine restrains adipogenesis of muscle Fibro/Adipogenic Progenitors from dystrophic mice by affecting AKT signaling
Source: Sci Rep. 2019 Mar 13;9:4360. doi: 10.1038/s41598-019-39538-y (PMC6416262; doi:10.1038/s41598-019-39538-y)
Supplement: Supplementary file 1 — The immunosuppressant drug azathioprine restrains adipogenesis of muscle Fibro/Adipogenic Progenitors from dystrophic mice by affecting AKT signaling [file 41598_2019_39538_MOESM1_ESM.pdf]

The immunosuppressant drug azathioprine restrains adipogenesis of muscle Fibro/Adipogenic Progenitors from dystrophic mice by affecting AKT signaling

Alessio Reggio[1], Filomena Spada[1], Marco Rosina[1], Giorgia Massacci[1], Alessandro Zuccotti[1], Claudia Fuoco[1], Cesare Gargioli[1], Luisa Castagnoli[1]* and Gianni Cesareni[1, 2]*

[1] Department of Biology, University of Rome "Tor Vergata", 00133 Rome, Italy

[2] Fondazione Santa Lucia Istituto di Ricovero e Cura a Carattere Scientifico (IRCCS), 00143 Rome, Italy

* castagnoli@uniroma2.it (LC)
* cesareni@uniroma2.it (GC)

Supplementary Materials and Methods

Muscle mononuclear cell differentiation

After four days from plating, muscle mononuclear cells were refreshed with new GM and cultured for additional two days before the induction of differentiation.

Fibrogenic differentiation was performed by exposing cells to GM supplemented by 5 ng/ml of human recombinant Transforming Growth Factor-β (TGF-β, PeproTech, catalog 100-21). Cells were treated for three days before fixing. Similarly, osteogenic differentiation was obtained by exposing cells to a fresh GM supplemented with 1 μg/ml of human recombinant Bone Morphogenetic Protein-2 (BMP-2, PeproTech, catalog #120-02). Treated cells were fixed after three days after morphogen exposure. Adipogenic differentiation was induced by exposing cells to the Adipocyte Differentiation Medium (ADM) obtained by adding 1 μg/ml human recombinant insulin (Sigma-Aldrich, catalog I9278), 0.5 mM 3-isobutyl-1-methylxanthine (IBMX, Sigma-Aldrich, catalog I5879), 1 μM Dexamethasone (Dexa, Sigma-Aldrich, catalog D4902) to GM. After 48 hours, the ADM was removed and replaced by the Adipocytes Maintenance Medium (AMM) obtained by adding 1 μg/ml insulin to GM. Muscle mononuclear cells were cultured in AMM for three additional days before fixing. Adipogenic differentiation was further stimulated by supplementing the ADM with 1 μM Rosiglitazone.

3T3-L1 preadipocytes culture and differentiation

3T3-L1 preadipocyte cell line was purchased from American Type Culture Collection (ATCC) and maintained in high glucose Dulbecco's Modified Eagle Medium (DMEM) (ATCC 30-2002) supplemented with 10% Iron Fortified Calf Serum (ATCC, catalog 30-2030). Differentiation assays were performed in 24-well plate (BD Falcon, catalog 353226) plating cells at the cell density of 3×10^4 cell/cm^2. Adipogenic differentiation was induced following ATCC guidelines. Briefly, three-days confluent cells were exposed to Adipocyte Differentiation Medium (ADM) (day 0 of differentiation). Two-days later, ADM was replaced with Adipocyte Maintenance Medium (AMM). AMM was refreshed every two days until complete cell differentiation (day 8 of differentiation). For the effects of drugs on the adipogenic differentiation, azathioprine (AZA) and trichostatin A (TSA) were added at the appropriate concentrations at day 0 or continuously for the whole differentiation period. For the effects on the signaling pathways perturbed by the drugs, a twelve-hours pre-treatment exposure was performed in growth medium and then followed by the treatment in ADM. Other experimental procedures on 3T3-L1 were performed with the same treatment protocols in 6-well plate (BD Falcon, catalog 353046) maintaining the relation cells/surface area.

Flow cytometry analysis

$CD45^-/CD31^-/\alpha7$-integrin$^-$ and $Sca1^+$ FAPs were routinely assessed for the expression of Platelet-Derived Growth Factor Receptor-α (PDGFRα). Briefly, purified FAPs were isolated and plated for four days in growth medium. After this period cell were dethatched from plastic using a scraper and washed in PBS 1X. 5×10^5 cells per ml were incubated for 30 minutes with the antibodies against CD140a (PDGFRα-APC, eBioscience, catalog 17-1401-81) and Sca1 (Ly-6A/E-FITC, BD Pharmigen, catalog 557405). Both antibodies were diluted 1:50 in FC buffer (1% BSA in PBS 1X) . Live/death cells were discriminated using propidium iodide (Pi). For each sample 10,000 events were acquired using BD FACSCALIBUR (BD Biosciences).

For cell cycle analysis, 3T3-L1 preadipocytes were cultured as described and the DNA content assessed by flow cytometry after Pi staining (BD Biosciences, BD FACSCALIBUR). Briefly, 3T3-L1 were induced to differentiate in ADM supplemented with AZA and TSA and the cells harvested at the indicated time points. Trypsinized cells were washed twice in PBS 1X, fixed and permeabilized for 30' in ice with a cold solution of methanol:acetone (4:1) in ratio 1:1 with PBS 1X. Fixed and permeabilized cells were centrifuged at 900×*g* and each pellet resuspended in a solution of 100 μg/ml ribonuclease A (Sigma-Aldrich, catalog R6513) in PBS 1X for 30 minutes at RT. Samples were stained with propidium iodide for 20 minutes at RT at the final concentration of 0.5 mg/ml. For each sample 10,000 events were acquired. All cell cycle data were analyzed using FlowJo 10 (treestar).

Alkaline phosphatase (ALP) activity assay

ALP activity was revealed by colorimetric assay using NBT/BCIP stock solution (Sigma-Aldrich, catalog 11681451001). Briefly, fixed cells were washed twice with PBS 1X and incubated for 15 minutes at room temperature with a staining solution in which the substrates 0.33 mg/ml of nitro-blue tetrazolium chloride (NBT) and 0.165 mg/ml of 5-bromo-4-chloro-3-indolyphosphate p-toluidine salt (BCIP) were diluted in AP Buffer (100 mM TRIS, 10 mM NaCl, 1 mM MgCl). Stained cells were washed twice with PBS 1X and counterstained using Hoechst 33342.

Multiplex gene expression analysis by QuantiGene® Plex Assay

The Adipogenic and fibrogenic fates of *mdx* FAPs were assessed at the transcriptional level by multiplex gene expression analysis. The assay is based on the branched DNA (bDNA) signal amplification coupled to the multi-analyte profiling beads (xMAP®) technologies to enable the simultaneous detection and quantitation of multiple RNA targets. The complete capture beads panel is reported in Supplementary Table S2. FAPs were cultured and treated as described with the reported concentrations of AZA and TSA. Differentiated cells were directly lysed, avoiding RNA purification,

in 96-well and 4-fold dilutions were performed from the undiluted samples as recommended. RNA hybridizations have been performed according to Affymetrix recommendations.

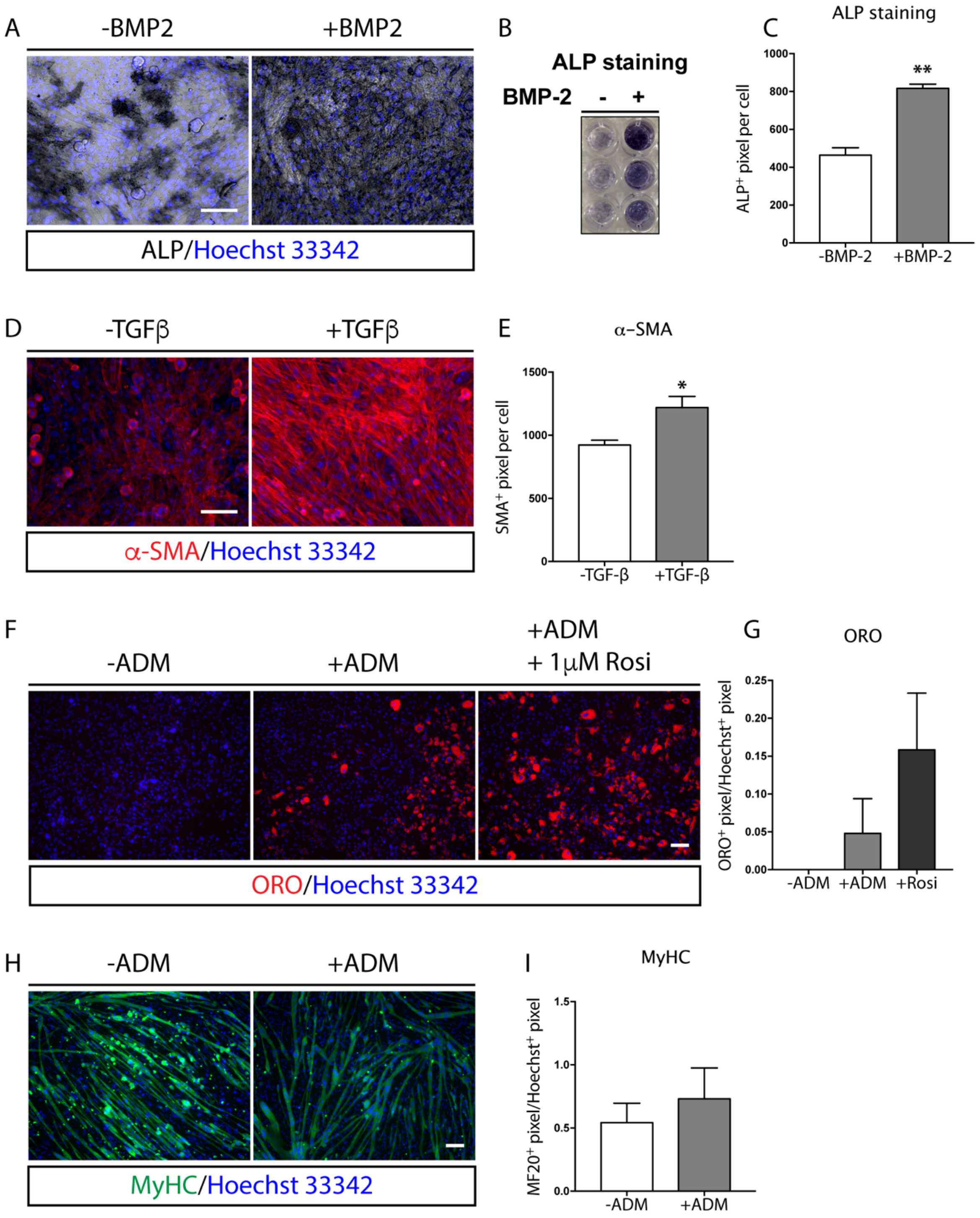
A
-BMP2
+BMP2
ALP/Hoechst 33342
B
ALP staining
BMP-2 - +
C
ALP staining
ALP+ pixel per cell
1000
800
600
400
200
0
**
-BMP-2
+BMP-2
D
-TGFβ
+TGFβ
α-SMA/Hoechst 33342
E
α-SMA
SMA+ pixel per cell
1500
1000
500
0
*
-TGF-β
+TGF-β
F
-ADM
+ADM
+ADM
+ 1μM Rosi
ORO/Hoechst 33342
G
ORO
ORO+ pixel/Hoechst+ pixel
0.25
0.20
0.15
0.10
0.05
0.00
-ADM
+ADM
+Rosi
H
-ADM
+ADM
MyHC/Hoechst 33342
I
MyHC
MF20+ pixel/Hoechst+ pixel
1.5
1.0
0.5
0.0
-ADM
+ADM

Supplementary Figure S1. **Differentiation potential of muscle mononuclear cells purified from wild type mice**. Muscle mononuclear cells were isolated from wild type mice and cultured for 6 days in growth medium (GM) prior to differentiation induction. For inducing osteogenic differentiation, muscle mononuclear cells were incubated for 3 days in GM supplemented with 1 μg/ml BMP-2 or vehicle alone. A. Representative immunofluorescence, taken at 20× magnification, of differentiated cells stained for alkaline phosphatase (ALP) with the NBT/BCIP solution (black). B. ALP stained wells (black) from three independent samples of muscle mononuclear cells exposed for three days to the vehicle or to 1 μg/ml BMP-2, respectively. C. The bar plot represents the average fraction of the ALP-positive area (expressed in pixel) per cell, in each field, in the two experimental conditions. D. Fibrogenic differentiation was induced by incubating muscle mononuclear cells for 3 additional days in GM supplemented with 5 ng/ml TGF-β or vehicle alone. Representative immunofluorescence, taken at 20× magnification, of differentiated α-SMA-positive myofibroblasts. E. Bar plot representing the average fraction of α-SMA-positive area (expressed in pixel) per cell, in each field, in the two experimental conditions. F. Muscle mononuclear cells were induced to differentiate into adipocytes by incubating cells in adipocyte induction medium (ADM) with or without 1 μM rosiglitazone (Rosi). Cells were incubated for three additional days in adipocyte maintenance medium (AMM). Representative immunostaining (10× magnification) of differentiated adipocytes (red) stained with the ORO solution. G. The bar plot represents the average ratio between the extent (expressed in pixel) of ORO-positive area normalized for the Hoechst 33342-positive area. H. Muscle mononuclear cells were induced to differentiate in the presence of ADM and assessed for their capability to differentiate into elongated myotubes. Representative immunofluorescence, taken at 10× magnification, of differentiated myotubes (green) immunostained with the anti-MF20 antibody. I. Bar plot represents the average ratio between the extent (expressed in pixel) of MF20-positive area and the Hoechst 33342-positive area, in each field. All values are means of at least three independent experiments ± SEM and the statistical significance was estimated by Student's *t*-test and defined as *$p < 0.05$; **$p < 0.01$; ***$p < 0.001$. Scale bar: (A, D, F, H) 100 μm.

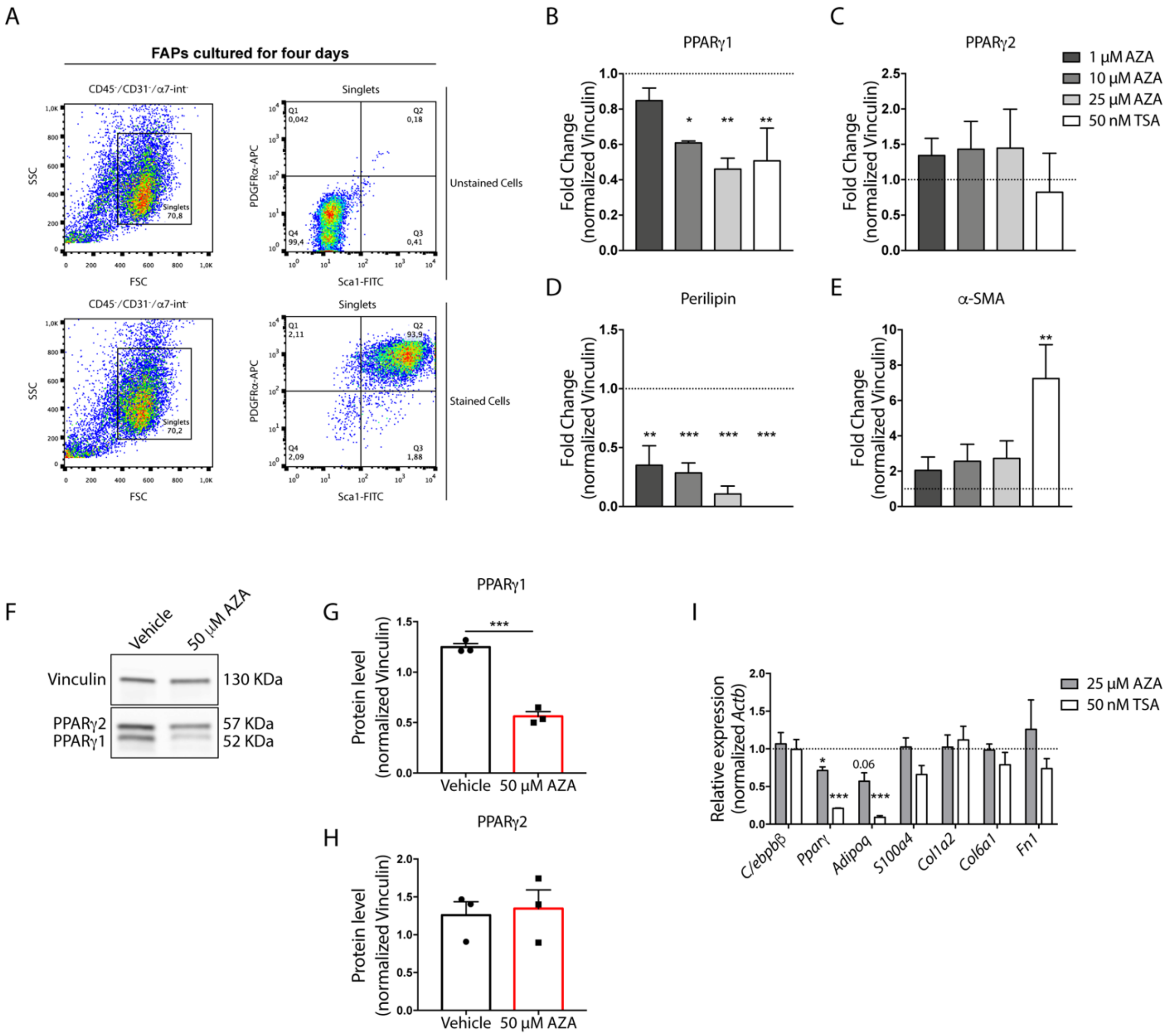

Supplementary Figure S2. **AZA impairs the upregulation of PPARγ1 and Perilipin without affecting α-SMA expression**. A. Scatter plots of FAPs plated in growth medium for four days and stained with antibodies raised against CD140a (PDGFRα-APC) and Sca1-FITC. B-E. Densitometric analysis of the western blot experiment shown in Figure 4 K. PPARγ1 (B) PPARγ2 (C) Perilipin (D) α-SMA (E). The protein signals were normalized over vinculin signal. Densitometric analysis were performed using ImageJ. All experimental data are presented as mean of three independent experiments ± SEM. The statistical significance was estimated by one way ANOVA and defined as $^{*}p < 0.05$; $^{**}p < 0.01$; $^{***}p < 0.001$. F. Representative immunoblot in which, for each sample, 15 μg of the protein lysate was electrophoresed on a 4-15% gradient gel and the protein levels of both PPARγ isoforms were revealed with a specific antibody. Vinculin serves as loading and normalizing control. The full-length blot is represented in Supplementary Figure S12. G, H. Bar plots of the densitometric analysis of PPARγ1(G) and PPARγ2(H) isoforms. Densitometric analysis were

performed using ImageJ. The experimental data is presented as mean of three independent experiments ± SEM. The statistical significance was estimated by Student's *t*-test and defined as $^{*}p < 0.05$; $^{**}p < 0.01$; $^{***}p < 0.001$. I. Freshly purified *mdx* FAPs were cultured and treated with 25 μM AZA and 50 nM TSA according to the protocol summarized in Figure 4 D to perform QuantiGene® plex analysis. Differentiated cells were lysed in 96-well and 4-fold dilutions were performed from the undiluted samples as recommended. Diluted cell lysates were incubated overnight at 54°C using the VorTemp 56 shaking incubator at 600 rpm in the presence of the capture beads that act as a support for the designed probe sets. The gene expression profile of *mdx* FAPs for adipogenic (*C/ebpβ*, *Pparγ*, *Adipoq*) and fibrogenic genes (*S100a4*, *Col1a2*, *Col6a1*, *Fn1*) was detected by the MagPIX® system. The assay was run in technical triplicates for two independent samples (*n*=2). The relative expression level for each gene arises from the mean fluorescence intensity (MFI) of the coloured bead-oligo associated upon normalization over the MFI of the bead of the *Actb* gene. Statistical significance was estimated by Student's *t*-test and defined as $^{*}p < 0.05$; $^{**}p < 0.01$; $^{***}p < 0.001$. The full list of each oligo and its cognate bead is reported in Supplementary Table S2.

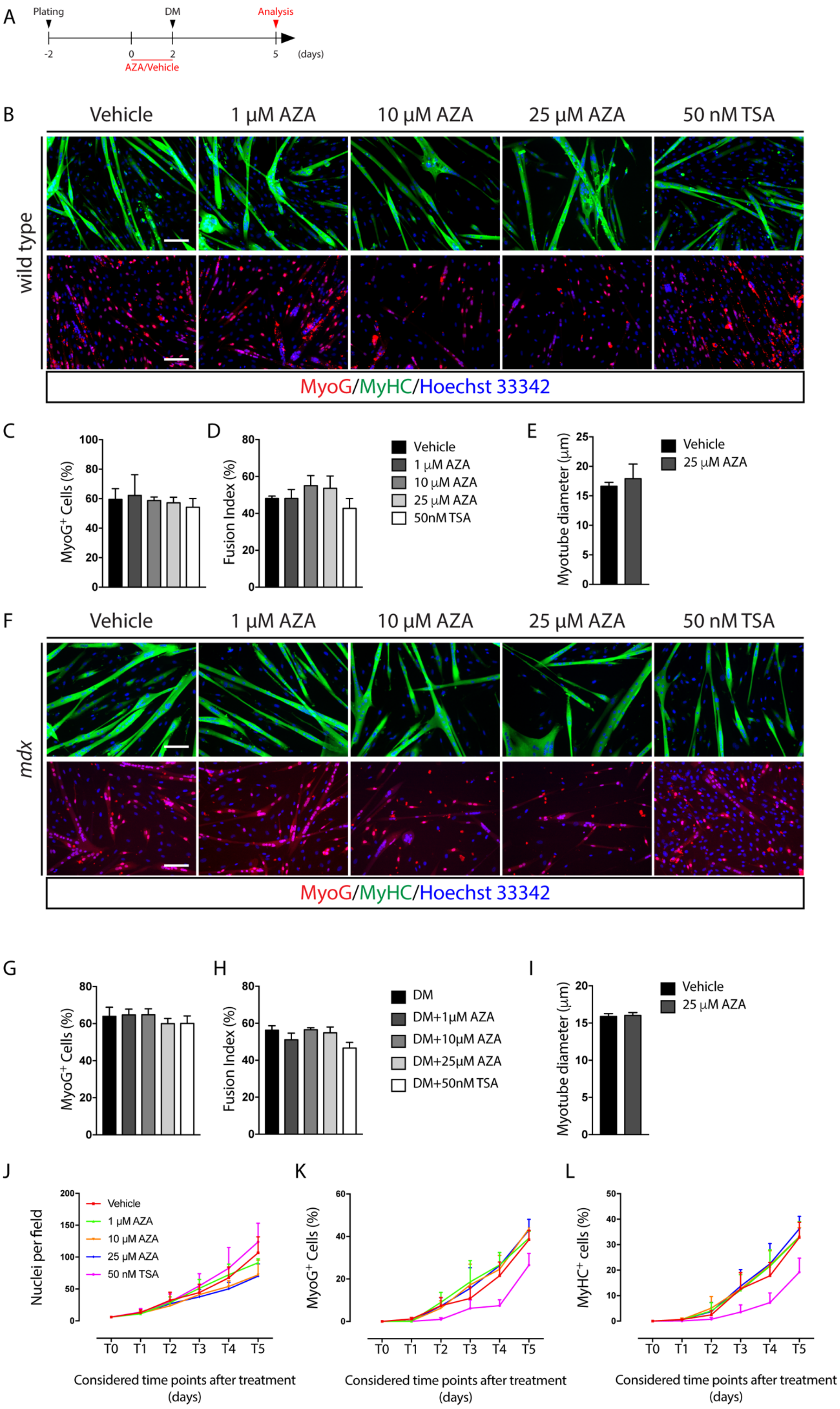
A
Plating
DM
Analysis
-2
0
2
5
(days)
AZA/Vehicle
B
Vehicle
1 μM AZA
10 μM AZA
25 μM AZA
50 nM TSA
wild type
MyoG/MyHC/Hoechst 33342
C
MyoG+ Cells (%)
D
Fusion Index (%)
Vehicle
1 μM AZA
10 μM AZA
25 μM AZA
50nM TSA
E
Myotube diameter (μm)
Vehicle
25 μM AZA
F
Vehicle
1 μM AZA
10 μM AZA
25 μM AZA
50 nM TSA
mdx
MyoG/MyHC/Hoechst 33342
G
MyoG+ Cells (%)
H
Fusion Index (%)
DM
DM+1μM AZA
DM+10μM AZA
DM+25μM AZA
DM+50nM TSA
I
Myotube diameter (μm)
Vehicle
25 μM AZA
J
Nuclei per field
Vehicle
1 μM AZA
10 μM AZA
25 μM AZA
50 nM TSA
T0 T1 T2 T3 T4 T5
Considered time points after treatment (days)
K
MyoG+ Cells (%)
T0 T1 T2 T3 T4 T5
Considered time points after treatment (days)
L
MyHC+ cells (%)
T0 T1 T2 T3 T4 T5
Considered time points after treatment (days)

Supplementary Figure S3. **AZA does not affect the myogenic potential of *in vitro* differentiating MuSCs**. A. Schematic representation of the differentiation protocol to induce myogenic differentiation of MuSCs *in vitro*. Freshly, purified MuSCs were cultured for two days in growth medium (SC-GM). Two days later, cells were treated with increasing concentrations (1, 10, 25 μM) of AZA and 50 nM TSA for 48 hours and then shifted in the differentiation medium (SC-DM) for three additional days. Differentiated MuSCs were immunostained to visualize elongated myotubes positive for MyHC (green) using an anti-MF20 antibody (upper panel). Alternatively, MuSCs were immunostained for myogenin (MyoG) (red). B. Representative immunofluorescence analysis, taken at 20× magnification, of differentiated wild type MuSCs in presence of increasing concentrations (1, 10, 25 μM) of AZA and 50 nM TSA. C-E. Bar graphs represent the average percentage of MyoG-positive cells (C), the average percentage of fusion index (D) and the average myotube thickness (E)between vehicle- and AZA-treated samples in each field. F. Representative immunofluorescence analysis, taken at 20× magnification, of differentiated *mdx* MuSCs in presence of increasing concentrations (1, 10, 25 μM) of AZA and 50 nM TSA. G-I. Bar graphs represent the average percentage of MyoG-positive cells (G), the average percentage of fusion index (H) and the average myotube thickness (I) between vehicle- and AZA-treated samples in each field. J-L. Line graphs represent the average number of nuclei (J), myogenin-positive cells (K) and MyHC-positive cells (L) per field along the considered time points after AZA treatment. All values are means of at least three independent experiments ± SEM. The statistical significance was estimated by one way ANOVA and defined as *$p < 0.05$; **$p < 0.01$; ***$p < 0.001$. Scale bar: (B, F) 100 μM.

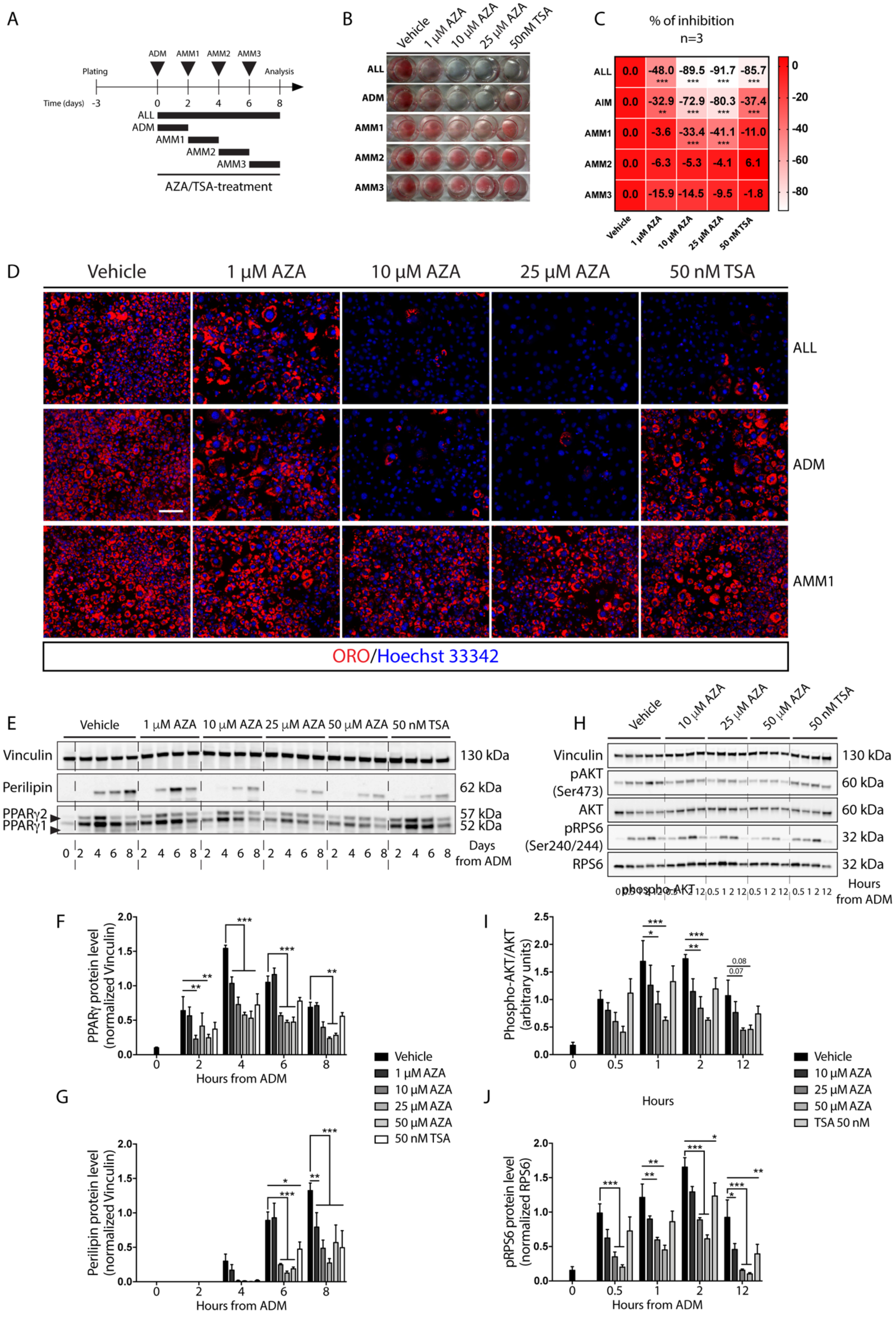

A
ADM
AMM1
AMM2
AMM3
Plating
Analysis
Time (days) -3 0 2 4 6 8
ALL
ADM
AMM1
AMM2
AMM3
AZA/TSA-treatment
B
Vehicle
1 µM AZA
10 µM AZA
25 µM AZA
50nM TSA
ALL
ADM
AMM1
AMM2
AMM3
C
% of inhibition
n=3
ALL 0.0 -48.0 -89.5 -91.7 -85.7
AIM 0.0 -32.9 -72.9 -80.3 -37.4
AMM1 0.0 -3.6 -33.4 -41.1 -11.0
AMM2 0.0 -6.3 -5.3 -4.1 6.1
AMM3 0.0 -15.9 -14.5 -9.5 -1.8
0
-20
-40
-60
-80
Vehicle
1 µM AZA
10 µM AZA
25 µM AZA
50 nM TSA
D
Vehicle
1 µM AZA
10 µM AZA
25 µM AZA
50 nM TSA
ALL
ADM
AMM1
ORO/Hoechst 33342
E
Vehicle
1 µM AZA
10 µM AZA
25 µM AZA
50 µM AZA
50 nM TSA
Vinculin
Perilipin
PPARγ2
PPARγ1
130 kDa
62 kDa
57 kDa
52 kDa
Days from ADM
H
Vehicle
10 µM AZA
25 µM AZA
50 µM AZA
50 nM TSA
Vinculin
pAKT (Ser473)
AKT
pRPS6 (Ser240/244)
RPS6
130 kDa
60 kDa
60 kDa
32 kDa
32 kDa
Hours from ADM
F
PPARγ protein level (normalized Vinculin)
Hours from ADM
G
Perilipin protein level (normalized Vinculin)
Hours from ADM
Vehicle
1 µM AZA
10 µM AZA
25 µM AZA
50 µM AZA
50 nM TSA
I
Phospho-AKT/AKT (arbitrary units)
Hours
0.08
0.07
J
pRPS6 protein level (normalized RPS6)
Hours from ADM
Vehicle
10 µM AZA
25 µM AZA
50 µM AZA
TSA 50 nM

Supplementary Figure S4. **AZA impairs *in vitro* adipogenesis of differentiating 3T3-L1 preadipocytes**. A. Schematic representation of the differentiation protocol used to induce the adipogenic differentiation of 3T3-L1 *in vitro*. B. 3T3-L1 cells were treated as illustrated in A with increasing concentrations (1, 10, 25 μM) of AZA or 50 nM TSA and stained with ORO. C. Heat map representing adipogenic inhibition in treated samples as estimated by comparing the optical densities at 495 nm of ORO stain. Multiple comparisons were estimated using two-way ANOVA with the Dunnet's correction. D. Representative immunofluorescence (20× magnification) of differentiating 3T3-L1 after treatment with 0, 1, 10, 25 μM of AZA and 50 nM TSA from the experiment shown in B-C. In the upper line, cells were treated with AZA and TSA over all the differentiation period while in the middle and the lower row, cells were treated for only 48 hours either in the ADM or the AMM1 period, respectively. Adipocytes (red) were stained with ORO solution and nuclei (blue) with Hoechst 33342. E. Representative western blot analysis of differentiating 3T3-L1 treated during the ADM period with increasing concentration of AZA (1, 10, 25, 50 μM) and 50 nM TSA. For each sample, 15 μg of the whole protein lysate was electrophoresed on a 4-20% gradient gel and the protein levels of perilipin and both PPARγ isoforms were revealed with specific antibodies. Vinculin serves as loading and normalizing control. Full-length blots are represented in Supplementary Figure S10. F, G. Bar graph represents the densitometric analysis of the protein level of PPARγ (F) or Perilipin (G) normalized over vinculin. H. Representative western blot analysis of a time course (0.5, 1, 2 and 12 hours) of differentiating 3T3-L1 treated during the ADM period with increasing concentration of AZA (10, 25, 50 μM) or 50 nM TSA. For each sample, 15 μg of the protein lysate was electrophoresed on a 4-20% gradient gel and the protein level of pAKT, AKT, pRPS6, RPS6 were revealed with specific antibodies. Vinculin serves as loading control. Full-length blots are represented in Supplementary Figure S11. I, J. Bar graph presents the densitometric analysis of the protein level of pAKT normalized over AKT (I) or pRPS6 normalized over RPS6 (J). All experimental values are means of at least three independent experiments ± SEM. The statistical significance was estimated by two-way ANOVA and defined as $^{*}p < 0.05$; $^{**}p < 0.01$; $^{***}p < 0.001$. Scale bar: (D) 100 μm.

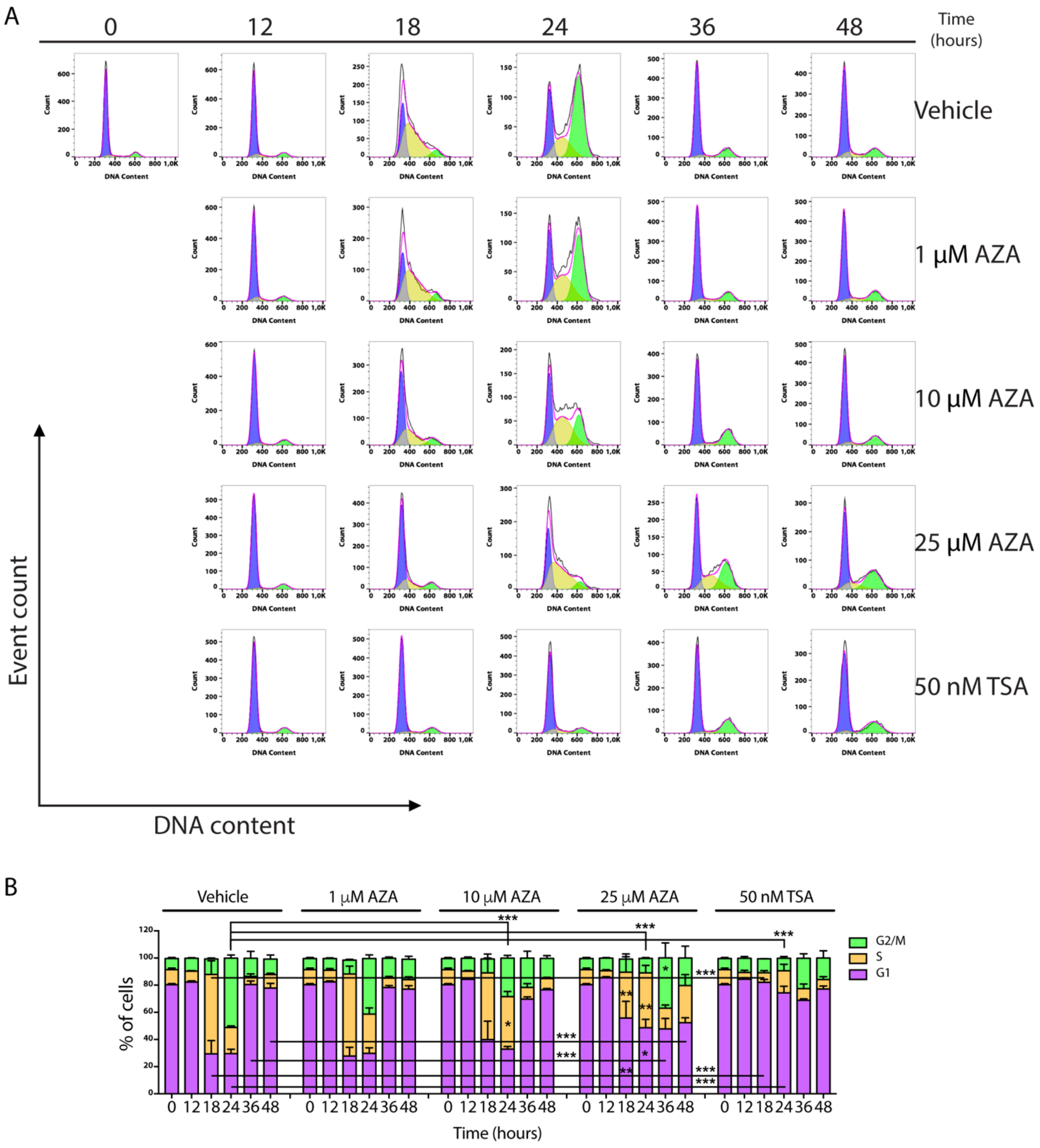
A
0
12
18
24
36
48
Time (hours)
Vehicle
1 μM AZA
10 μM AZA
25 μM AZA
50 nM TSA
Count
DNA Content
Event count
DNA content
B
Vehicle
1 μM AZA
10 μM AZA
25 μM AZA
50 nM TSA
G2/M
S
G1
% of cells
Time (hours)

Supplementary Figure S5. **AZA impairs MCE in differentiating 3T3-L1 preadipocytes**. A. Representative cytofluorigram describing the cell cycle phase kinetics of differentiating 3T3-L1 in the presence of DMSO (vehicle) and increasing concentrations (1, 10, 25 μM) of AZA or 50 nM TSA. G0/G1 (purple), S (yellow) and G2/M (green). B. Bar plot of the percentage of cells in each cell cycle phase for vehicle and treated samples. All experimental values are means of at least three independent experiments ± SEM and the statistical significance was estimated by two-way ANOVA and defined as *$p < 0.05$; **$p < 0.01$; ***$p < 0.001$.

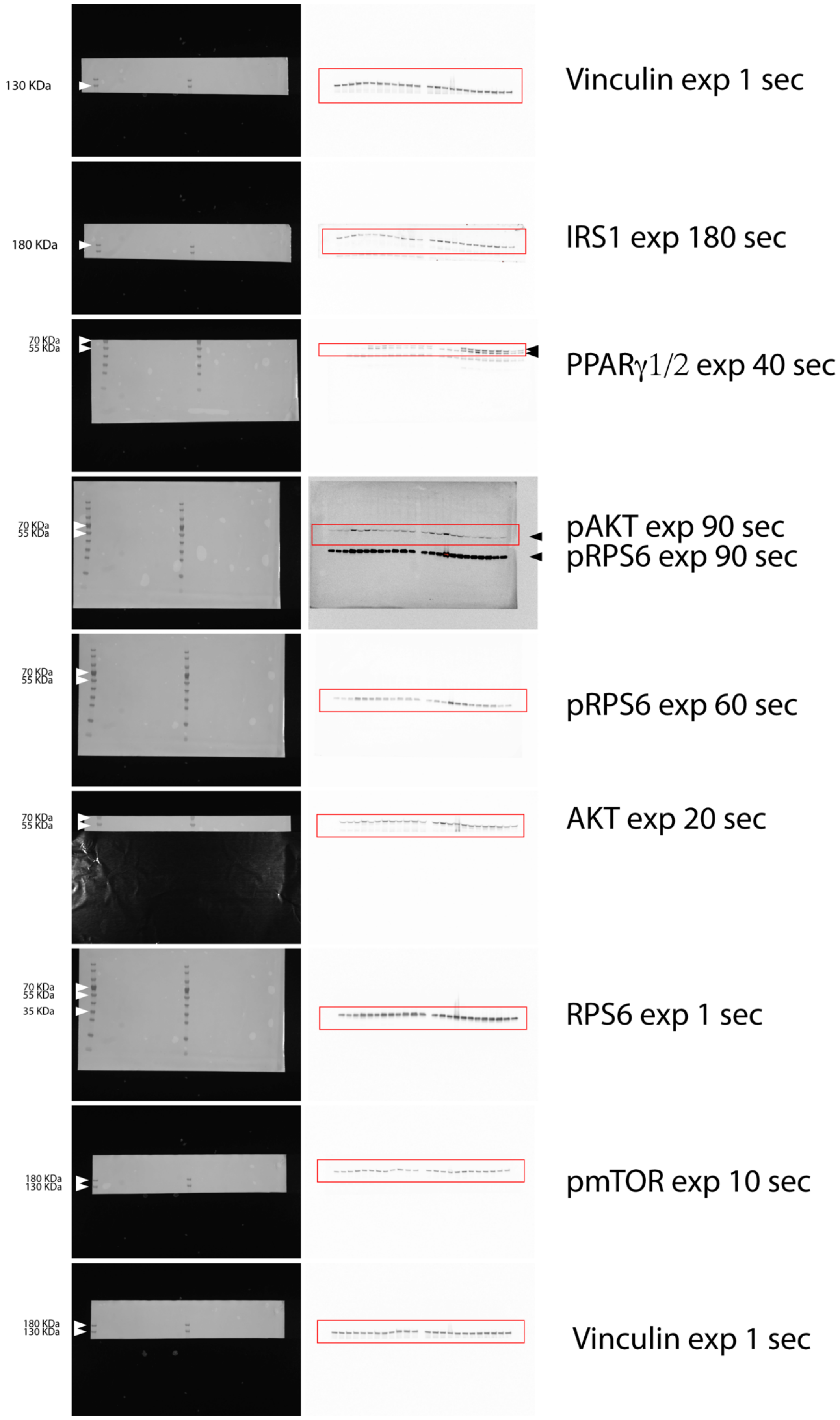

Supplementary Figure S6. **Full-length gel of Fig. 3 A**.

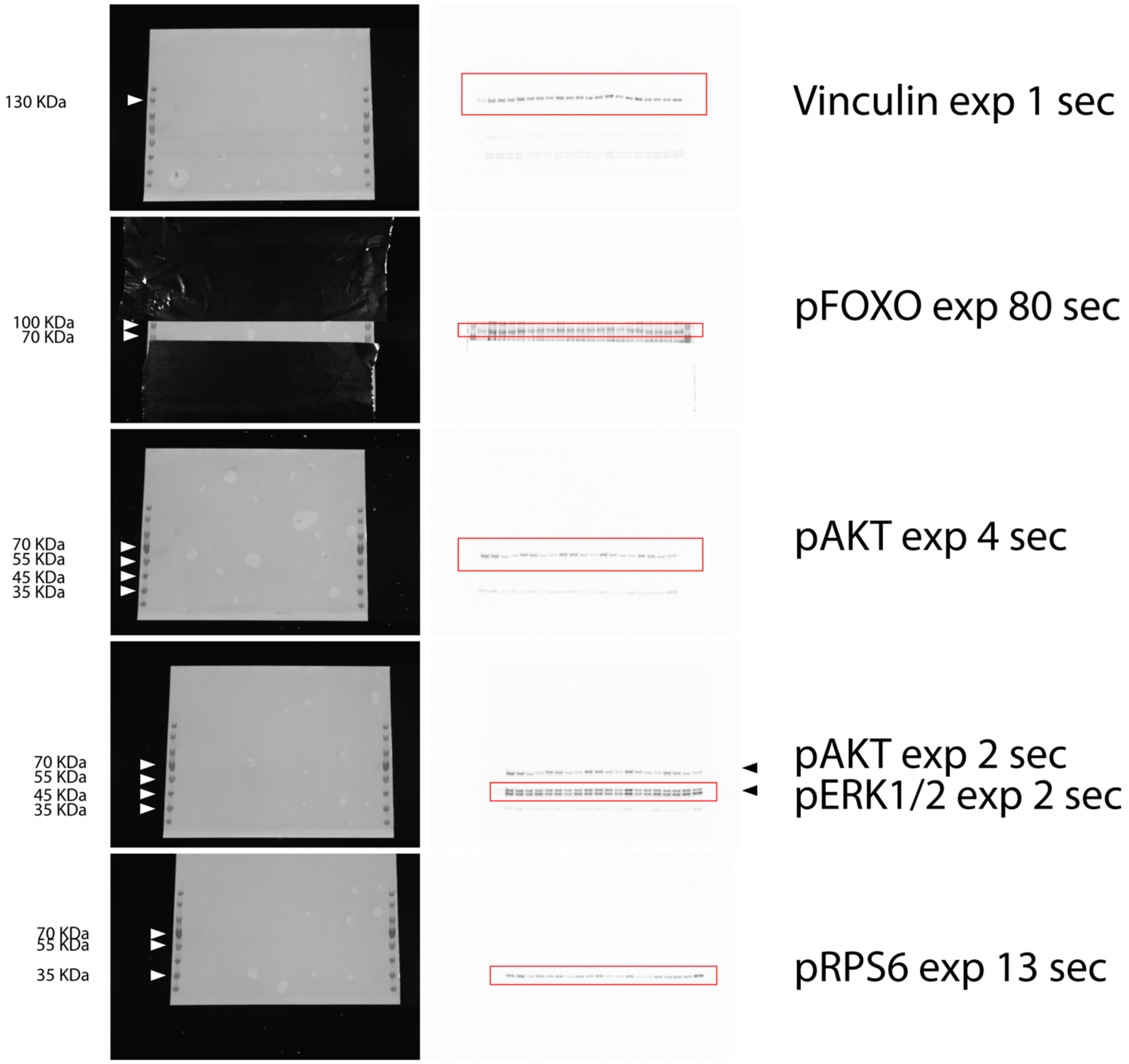

Supplementary Figure S7. **Full-length gel of Fig. 4 B**.

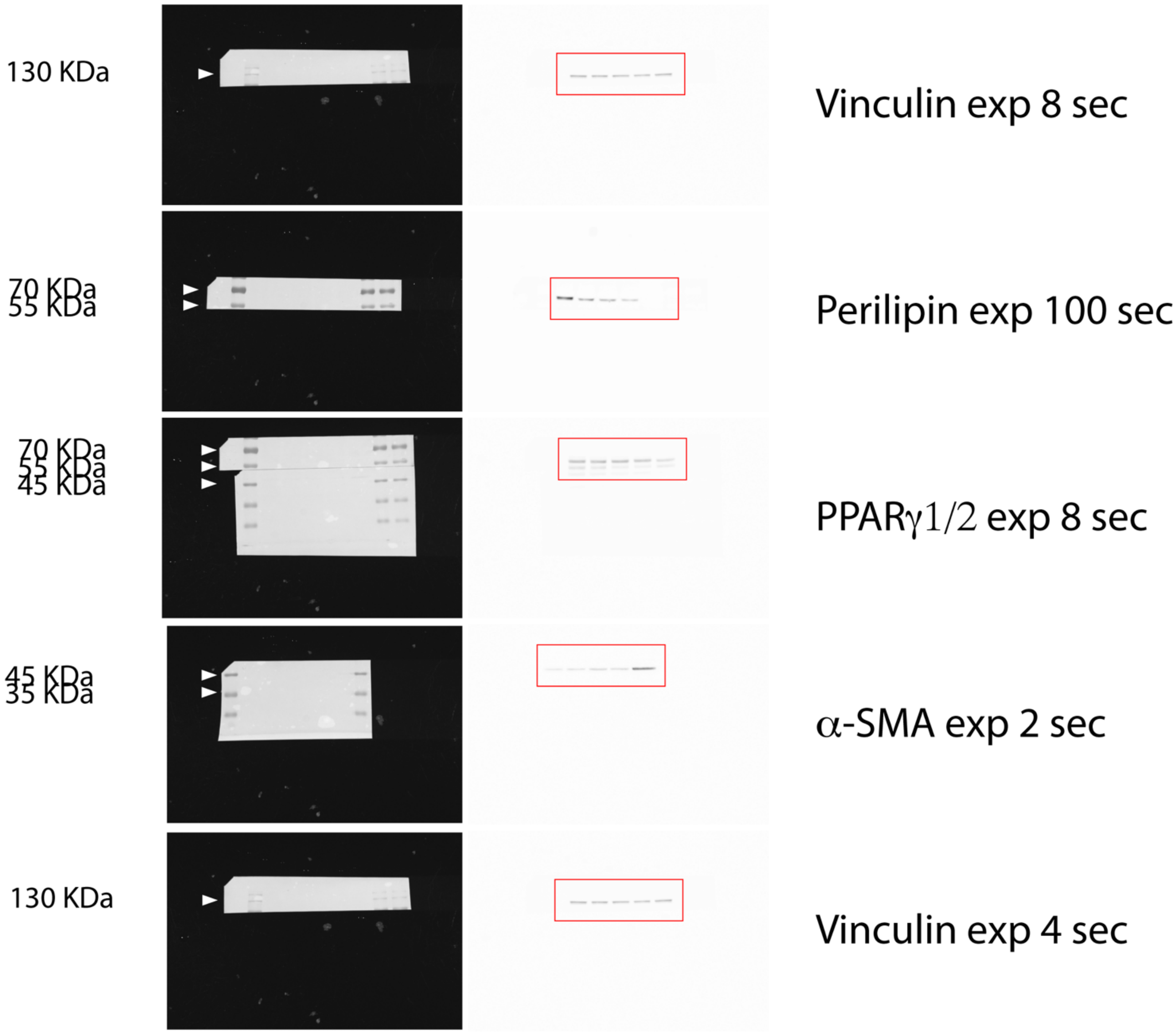

Supplementary Figure S8. **Full-length gel of Fig. 4 K**.

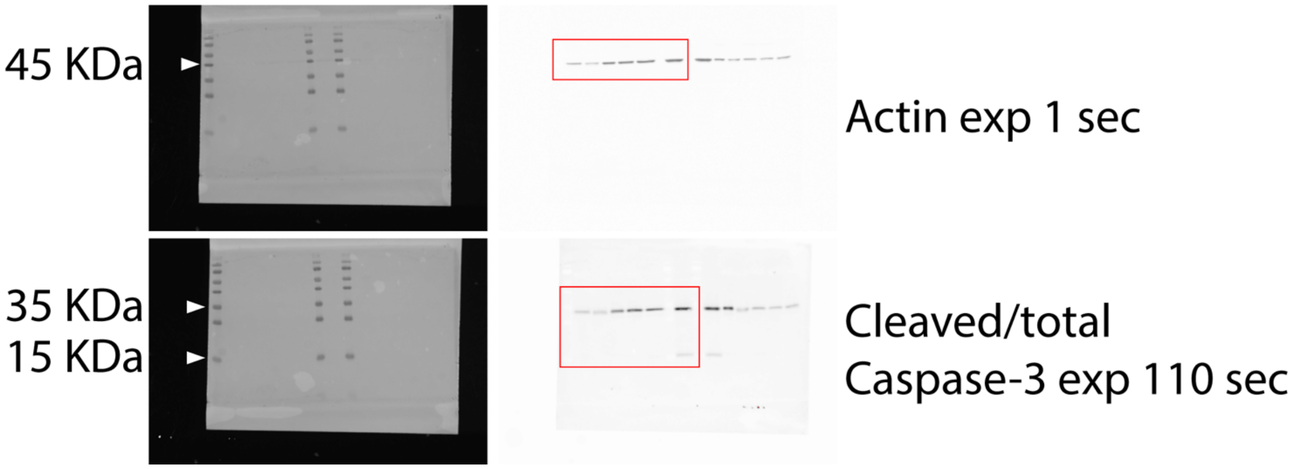

Supplementary Figure S9. **Full-length gel of Fig. 5 G**.

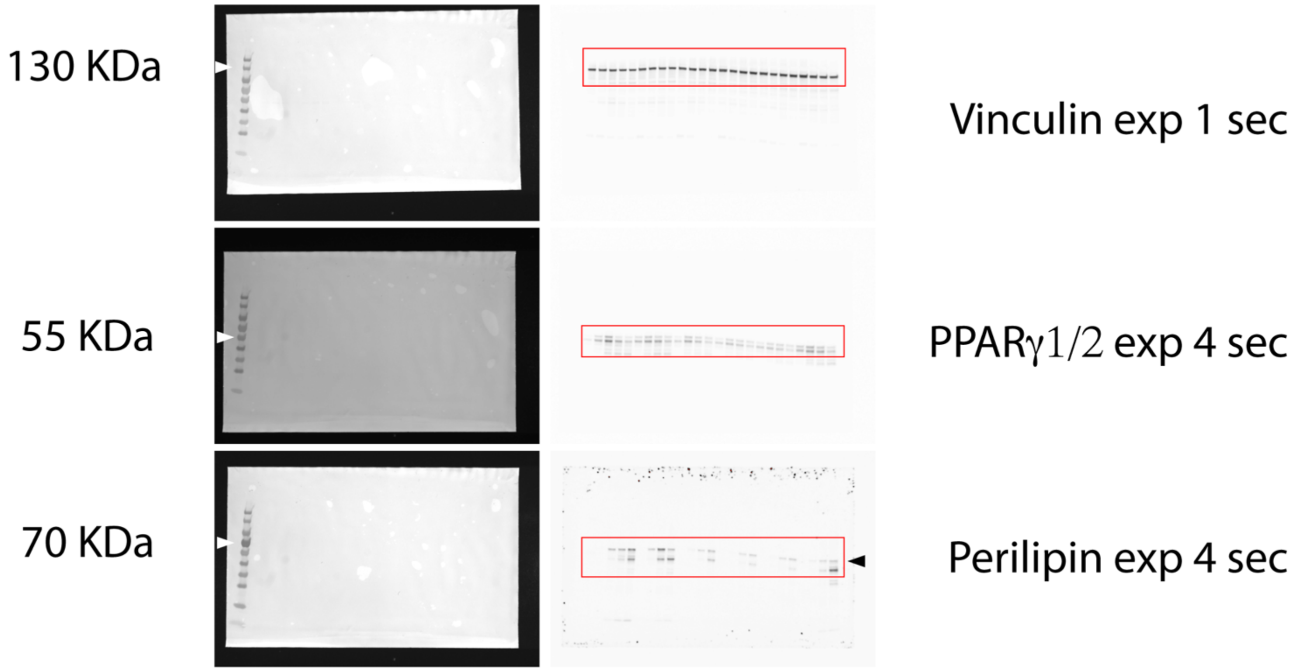

Supplementary Figure S10. **Full-length gel of Supplementary Figure S4 E**.

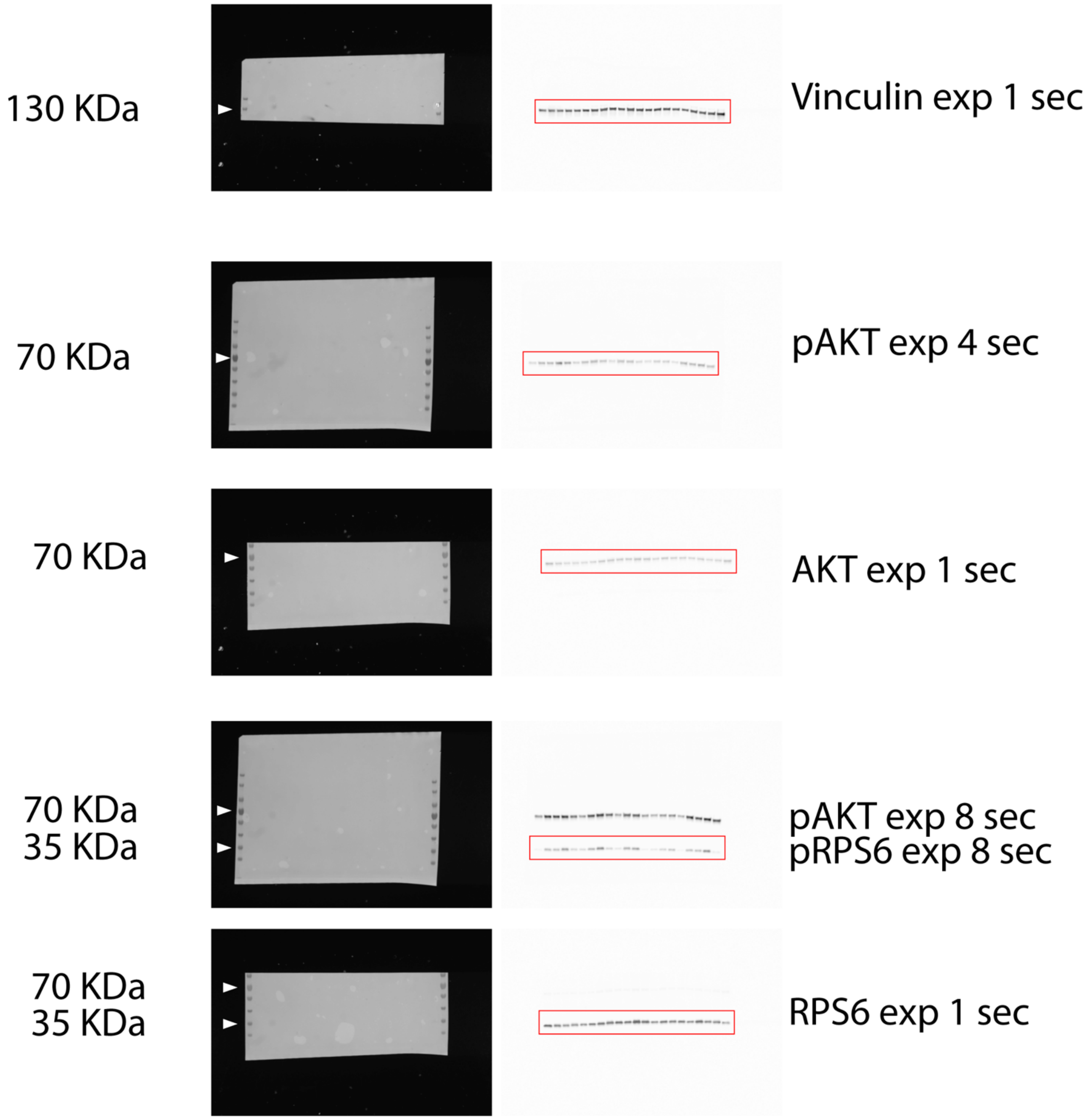

Supplementary Figure S11. **Full-length gel of Supplementary Figure S4 H**.

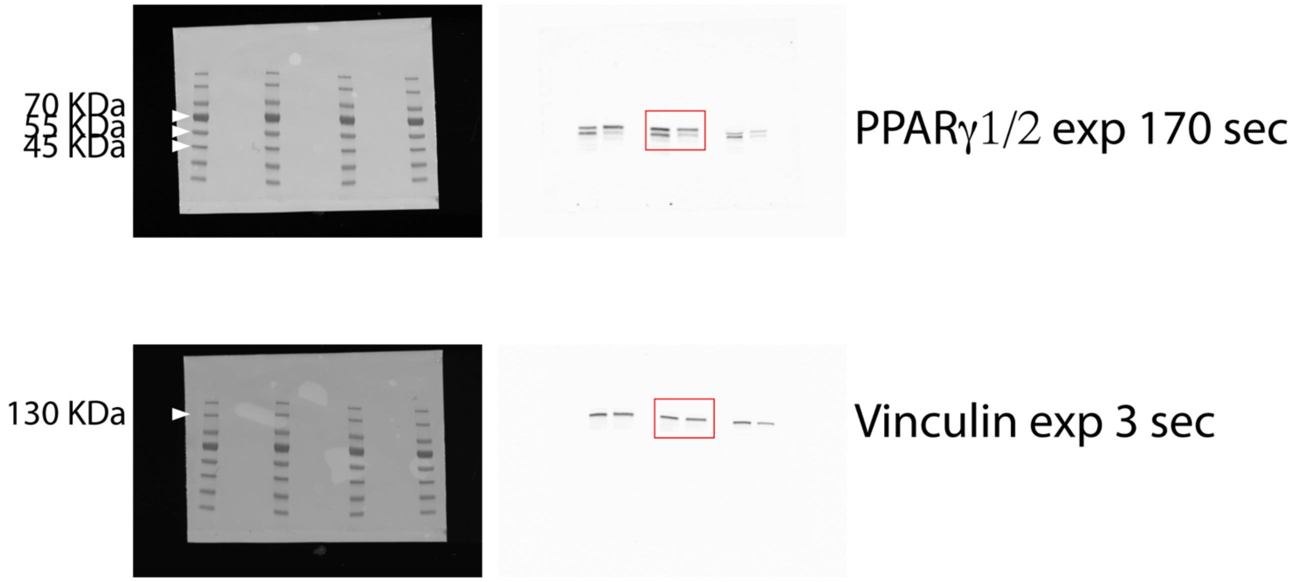

Supplementary Figure S12. **Full-length gel of Supplementary Figure S2 F**.

			Dilution	
Antibody	Vendor	Catalog number	Immunoblotting	Immunofluorescence
Perilipin (D418)	CST	3470	1:1000	
PPARg (81B8)	CST	2443	1:1000	1:50
pAKT (Ser473) (D9E)XP®	CST	4060	1:4000	
AKT	CST	9272	1:1000	
pRPS6 (Ser202/204)	CST	2215	1:5000	
RPS6 (5G10)	CST	2217	1:1000	
pmTOR (Ser2448) (D9C2)XP®	CST	5536	1:2000	
IRS-1	Upstate	06-248	1:500	
pFOXO1 (Ser256)	CST	9461	1:3000	
p-p44/42 (pERK1/2) (Thr202/Tyr204)	CST	90101	1:4000	
α-Smooth Muscle Actin (α-SMA) (1A4)	Sigma Aldrich	A5228	1:1000	1:300
Vinculin (spm227)	Abcam	ab18058	1:2000	
Caspase-3	CST	9662	1:1000	
MF20	SDHB	319-335-3826	1:1000	1:200
Myogenin (F5D)	eBioscience	14-5643-80	1:1000	1:300
Anti-mouse HRP	Bio-Rad	1721011	1:3000	
Anti-rabbit HRP	Bio-Rad	1706515	1:3000	
Anti-mouse Alexa Fluor® 488	Molecular Probe	A11001		1:200
Anti-mouse Alexa Fluor® 555	Molecular Probe	A21425		1:200
Anti-rabbit Alexa Fluor® 488	ThermoFisher Scientific	A11008		1:200

Supplementary Table S1. **List of the antibodies used in this study**.

Bead Number	Target symbol	Target name
18	***Cebpb***	CCAAT/enhancer binding protein (C/EBP) beta
30	***Col1a2***	Procollagen, type I, alpha 2
36	***Adipoq***	Adipocyte C1Q and collagen domain containing
45	***Pparg***	Peroxisome proliferator activated receptor gamma
46	***Col6a1***	Procollagen, type VI, alpha 1
48	***Actb***	Actin, beta, cytoplasmic
55	***Fn1***	Fibronectin

Supplementary Table S2. **Panel adopted for the QuantiGene® Plex Assay.**
